# The oral glucose tolerance test-derived incremental glucose peak is associated with greater arterial stiffness and maladaptive arterial remodeling: The Maastricht Study

**DOI:** 10.1186/s12933-019-0950-x

**Published:** 2019-11-14

**Authors:** Yuri D. Foreman, Martijn C. G. J. Brouwers, Tos T. J. M. Berendschot, Martien C. J. M. van Dongen, Simone J. P. M. Eussen, Marleen M. J. van Greevenbroek, Ronald M. A. Henry, Alfons J. H. M. Houben, Carla J. H. van der Kallen, Abraham A. Kroon, Koen D. Reesink, Miranda T. Schram, Nicolaas C. Schaper, Coen D. A. Stehouwer

**Affiliations:** 1 Present Address: Department of Internal Medicine, Maastricht University Medical Center+, Maastricht, The Netherlands; 2 CARIM School for Cardiovascular Diseases, Maastricht University, Maastricht, The Netherlands; 3 Department of Internal Medicine, Division of Endocrinology and Metabolic Disease, Maastricht University Medical Center+, Maastricht, The Netherlands; 4 University Eye Clinic Maastricht, Maastricht University Medical Center+, Maastricht, The Netherlands; 5 Department of Epidemiology, Maastricht University, Maastricht, The Netherlands; 6 CAPHRI Care and Public Health Research Institute, Maastricht University, Maastricht, The Netherlands; 7 Heart and Vascular Center, Maastricht University Medical Center+, Maastricht, The Netherlands; 8 Department of Biomedical Engineering, Maastricht University, Maastricht, The Netherlands

**Keywords:** Arterial remodeling, Arterial stiffness, Glucose metabolism status, Glucose variability, Oral glucose tolerance test

## Abstract

**Background:**

Daily glucose variability may contribute to vascular complication development irrespective of mean glucose values. The incremental glucose peak (IGP) during an oral glucose tolerance test (OGTT) can be used as a proxy of glucose variability. We investigated the association of IGP with arterial stiffness, arterial remodeling, and microvascular function, independent of HbA_1c_ and other confounders.

**Methods:**

IGP was calculated as the peak minus baseline plasma glucose value during a seven-point OGTT in 2758 participants (age: 60 ± 8 years; 48% women) of The Maastricht Study, an observational population-based cohort. We assessed the cross-sectional associations between IGP and arterial stiffness (carotid-femoral pulse wave velocity [cf-PWV], carotid distensibility coefficient [carDC]), arterial remodeling (carotid intima-media thickness [cIMT]; mean [CWS_mean_] and pulsatile [CWS_puls_] circumferential wall stress), and microvascular function (retinal arteriolar average dilatation; heat-induced skin hyperemia) via multiple linear regression with adjustment for age, sex, HbA_1c_, cardiovascular risk factors, lifestyle factors, and medication use.

**Results:**

Higher IGP was independently associated with higher cf-PWV (regression coefficient [B]: 0.054 m/s [0.020; 0.089]) and with higher CWS_mean_ (B: 0.227 kPa [0.008; 0.446]). IGP was not independently associated with carDC (B: − 0.026 10^−3^/kPa [− 0.112; 0.060]), cIMT (B: − 2.745 µm [− 5.736; 0.245]), CWS_puls_ (B: 0.108 kPa [− 0.054; 0.270]), retinal arteriolar average dilatation (B: − 0.022% [− 0.087; 0.043]), or heat-induced skin hyperemia (B: − 1.380% [− 22.273; 19.513]).

**Conclusions:**

IGP was independently associated with aortic stiffness and maladaptive carotid remodeling, but not with carotid stiffness, cIMT, and microvascular function measures. Future studies should investigate whether glucose variability is associated with cardiovascular disease.

## Background

Chronic hyperglycemia is a key factor in the development of type 2 diabetes-related macrovascular and microvascular complications [1, 2]. In the macrovasculature, elevated mean blood glucose levels contribute to arterial stiffening [3, 4], atherosclerosis [1], and large artery endothelial dysfunction [5]. In the microvasculature, hyperglycemia and endothelial dysfunction are considered to be bidirectionally related, potentially entering a vicious cycle that could lead to microvascular complications [6]. Of note, these pathophysiologic processes have been shown to already occur in the prediabetic state [7, 8].

Importantly, chronic hyperglycemia per se does not fully explain the incidence of complications [9]. Daily glucose variability could play a role in vascular complication development irrespective of mean glucose values [10]. While relatively small observational studies have found conflicting results regarding the association between glucose variability and classic diabetic complications [11–13], experimental studies have shown that greater glucose variability can be harmful independent of mean glucose values [14, 15].

Continuous glucose monitoring, the gold standard for glucose variability assessment [16], is a challenging technology to use in a large epidemiological setting. The incremental glucose peak (IGP), i.e. the glucose increase from baseline during an oral glucose tolerance test (OGTT), can be used as an index of glucose variability [17]. In view of the aforementioned, we investigated, in a large population-based cohort, whether IGP is associated with arterial stiffness, arterial remodeling, and microvascular function, independent of HbA_1c_.

## Methods

### Study population and design

We used data from The Maastricht Study, an observational prospective population-based cohort study. The rationale and methodology have been described previously [18]. In brief, The Maastricht Study focuses on the etiology, pathophysiology, complications and comorbidities of type 2 diabetes, and is characterized by an extensive phenotyping approach. All individuals aged between 40 and 75 years and living in the southern part of the Netherlands were eligible for participation. We recruited participants through mass media campaigns and from the municipal registries and the regional Diabetes Patient Registry via mailings. For reasons of efficiency, we stratified recruitment according to known type 2 diabetes status, with an oversampling of individuals with type 2 diabetes. The present report includes cross-sectional data from the first 3451 participants who completed the baseline survey between November 2010 and September 2013. All examinations were performed within a three-month time window; the OGTT and vascular measurements were performed during different research visits. The Maastricht Study has been approved by the institutional medical ethical committee (NL31329.068.10) and the Minister of Health, Welfare and Sports of the Netherlands (Permit 131088-105234-PG). All participants gave written informed consent.

### Assessment of glucose metabolism status and incremental glucose peak

Participants underwent a standardized 2-h 75 g OGTT after fasting overnight to determine glucose metabolism status (GMS), which was defined according to the World Health Organization 2006 criteria as normal glucose metabolism (NGM), impaired fasting glucose, impaired glucose tolerance (combined as prediabetes), or type 2 diabetes [19]. For safety reasons, participants using insulin or with a fasting plasma glucose (FPG) value above 11.0 mmol/L (determined by finger prick) did not undergo the OGTT. For these individuals, we used FPG and information about their diabetes medication to determine GMS. During the OGTT, we took venous blood glucose samples at baseline and 15, 30, 45, 60, 90 and 120 min; we calculated IGP by subtracting FPG from the absolute glucose peak (AGP) value.

### Assessment of arterial stiffness, intima-media thickness and circumferential wall stress

The rationale and methodology of the macrovascular measurements have been described previously [20, 21]. We determined carotid-femoral pulse wave velocity (cf-PWV) with the use of applanation tonometry (SphygmoCor, Atcor Medical, Sydney, Australia) [22], and used the median of three consecutive cf-PWV recordings in our analyses.

We measured the left common carotid artery using an ultrasound scanner equipped with a 7.5-MHz linear probe (MyLab 70, Esaote Europe B.V., Maastricht, the Netherlands) to assess local carotid distension, intima-media thickness (cIMT), and interadventitial diameter (IAD) [23]. We quantified local arterial stiffness by calculating the carotid distensibility coefficient (carDC) based on the following formula: carDC = (2*ΔD*IAD + ΔD^2^)/(braPP*IAD^2^), where IAD is interadventitial arterial diameter, ΔD distension, and braPP brachial pulse pressure [24].

We defined cIMT as the distance between the lumen-intima and media-adventitia interfaces of the far (posterior) wall [23], and IAD as the distance between the media-adventitia interfaces of the near and far wall. The median carDC, cIMT and IAD of three consecutive measurements were used. We calculated carotid lumen diameter (LD) according to the following formula [25]: LD = IAD − (2*cIMT). In parallel with the vascular measurements, we determined mean heart rate (HR) and mean arterial pressure (MAP) every 5 min with an oscillometric device (Accutorr Plus, Datascope Inc., Montvale, NJ, USA). Mean (CWS_mean_) and pulsatile (CWS_puls_) carotid circumferential wall stress were calculated using the Lamé equation as CWS_mean_ = (MAP*(LD/2))/cIMT and CWS_puls_ = (carPP*(LD/2))/cIMT, where carotid pulse pressure (carPP) was obtained from carotid pressure waveform calibration [20].

### Assessment of microvascular function

The rationale and methodology of assessing the microcirculation of the retina and skin have been described previously [8]. In short, we measured the retinal microvascular dilation response to flicker light during a 50-s baseline, 40-s flicker-light provocation, and 60-s recovery phase, by use of the Dynamic Vessel Analyzer (DVA; Imedos, Jena, Germany). The integrated DVA software (version 4.51; Imedos) automatically calculated average baseline diameter size (expressed in measurement units; MUs) during the 20–50 s of baseline recording, and percentage dilation at time points 10 and 40 s during the flicker stimulation period. Two regression lines were drawn (at the 0–10-s and 10–40-s intervals) and averaged to assess average percentage dilation. We measured skin blood flow with a laser-Doppler system (Periflux 5000; Perimed, Järfalla, Sweden) equipped with a thermostatic laser-Doppler probe (PF457; Perimed) at the dorsal side of the left wrist. After a 2-min baseline recording, the probe temperature was rapidly increased to 44 °C and kept constant until the end of the registration. The heat-induced skin hyperemic response was expressed as the percentage increase in average perfusion units (PUs) during the 23-min heating phase over the average baseline PU.

### Measurement of covariates

As described previously [18], we assessed history of cardiovascular disease (CVD), physical activity, and smoking status (never, former, current) by questionnaire; calculated Mediterranean diet adherence according to Trichopoulou et al. based on a food frequency questionnaire [26]; assessed lipid-modifying, antihypertensive, and glucose-lowering medication use as part of a medication interview; measured weight, height, body mass index (BMI), and waist circumference, during a physical examination; measured office and 24-h ambulatory blood pressure (BP); measured HbA_1c_, fasting plasma insulin and lipid profile in fasting venous blood samples; quantified insulin resistance (IR) based on the updated Homeostatic Model Assessment (HOMA2-IR); measured albumin excretion in two 24-h urine collections; calculated the estimated glomerular filtration rate (eGFR) based on both serum creatinine and cystatin C [27]; and assessed retinopathy presence in both eyes via fundus photography.

### Statistical analysis

Normally distributed data are presented as mean and standard deviation (SD), non-normally distributed data as median and interquartile range (IQR), and categorical data as n (%). We used multivariable linear regression to study the associations between IGP and arterial stiffness (cf-PWV, carDC), arterial remodeling (cIMT, CWS_mean_, CWS_puls_), and microvascular function (retinal arteriolar average dilatation, heat-induced skin hyperemia). Model 1 was the crude model, which included only IGP as a determinant; model 2 was adjusted for age and sex; model 3 was additionally adjusted for HbA_1c_; model 4 was additionally adjusted for MAP (or alternatively office systolic BP; or carPP) and HR in case of cf-PWV only; model 5 was additionally adjusted for cardiovascular risk and lifestyle factors (i.e. BMI, smoking status, physical activity, Mediterranean diet score, antihypertensive and lipid-modifying drug use, fasting triglycerides and total-to-high-density lipoprotein cholesterol levels). The results are presented as: regression coefficient (B) (corresponding 95% confidence interval [CI]), *P* value. We considered a *P* value of < 0.05 statistically significant. To test the robustness of our findings, we performed multiple sensitivity analyses by: (1) additionally adjusting for history of CVD, retinopathy, eGFR, and urinary albumin excretion; (2) additionally adjusting for fasting plasma insulin or HOMA2-IR; (3) replacing HbA_1c_ with GMS or FPG; (4) replacing IGP with AGP or percentage increase from baseline (IGP_percentage_ = IGP/FPG*100%); (5) adjusting for alternative BP measurements (e.g. ambulatory 24-h systolic BP); and (6) replacing IGP with time to glucose peak. We incorporated interaction terms in the fully adjusted regression models to test for interactions between IGP and sex [28], as well as IGP and age, as previously advocated [29]. We considered a *P* value for interaction of < 0.10 statistically significant. We performed all statistical analyses with the Statistical Package for Social Sciences (Version 25.0; IBM, Chicago, IL).

## Results

### Study population characteristics

**Fig. 1:**
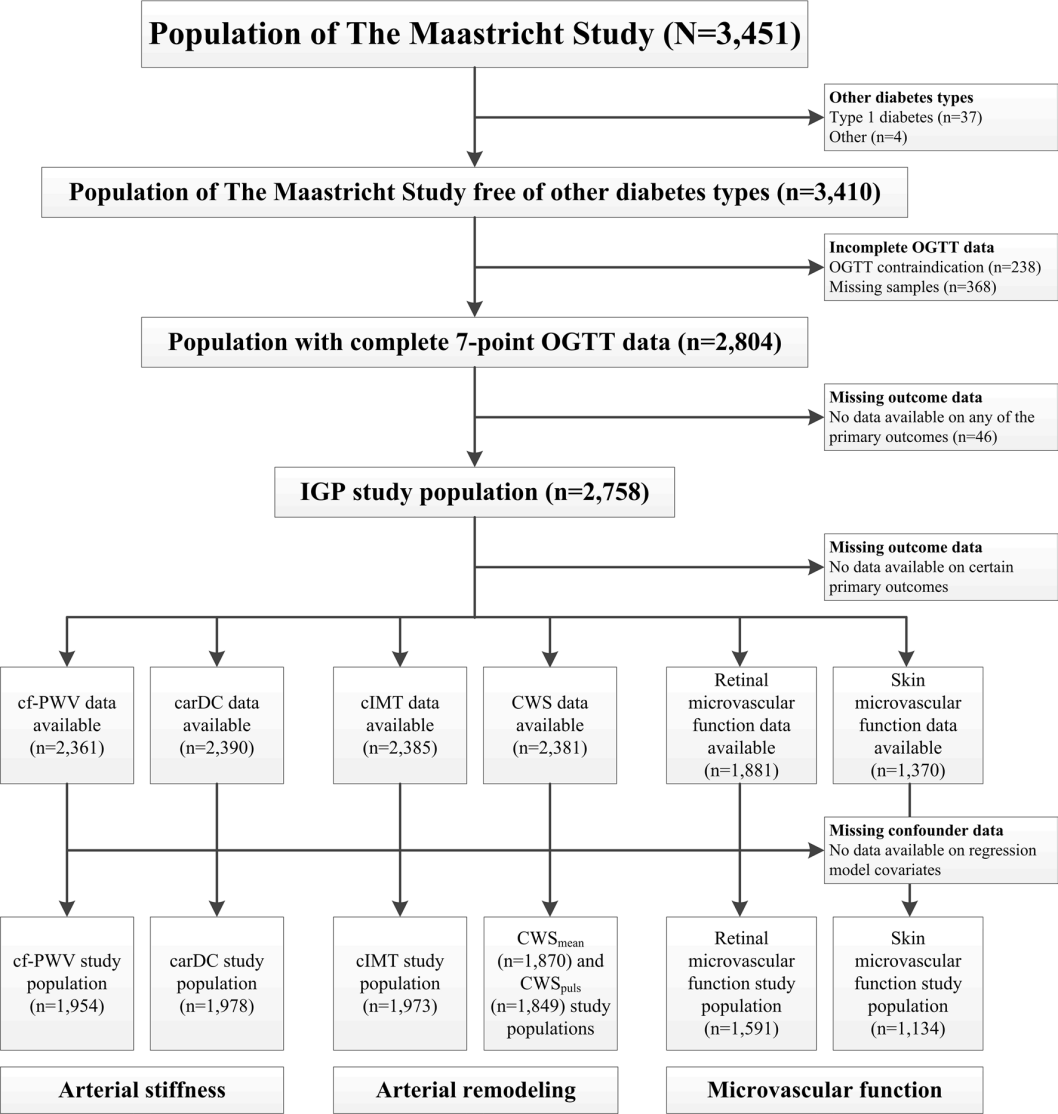
Flowchart of the IGP study population selection process. *OGTT* oral glucose tolerance test, *IGP* incremental glucose peak, *cf-PWV* carotid-femoral pulse wave velocity, *carDC* carotid distensibility coefficient, *cIMT* carotid intima-media thickness, *CWS*_*mean*_ mean circumferential wall stress, *CWS*_*puls*_ pulsatile circumferential wall stress

The total study population comprised 3451 individuals, from which we excluded 41 participants with diabetes types other than type 2 diabetes. Some participants had incomplete data on the seven-point OGTT, either because of missing glucose samples (n = 368) or an OGTT contraindication (n = 238; i.e. insulin use or plasma glucose levels > 11.0 mmol/L before initiation of the OGTT), resulting in a study population of 2804 individuals. Those with missing glucose samples were generally comparable to the final study population (Additional file 1: Table S1); as expected, those with an OGTT contraindication differed statistically significantly from the final study population with regard to almost all characteristics (Additional file 1: Table S1). Finally, for 46 participants all outcome data was missing. These individuals were similar to the final study population (Additional file 1: Table S1), which consisted of 2758 individuals. Since outcome and covariate data could not be obtained in all individuals (Additional file 1: Table S2), the number of individuals included in the different regression analyses varied (n = 1134–1978) (Fig. 1).

Table 1 shows the general characteristics of the final study population, stratified according to IGP tertiles. Participants in the highest tertile were older, predominantly male, and had a worse cardiometabolic profile, i.e. higher BMI, waist circumference, systolic BP, and fasting glucose, 2-h post-load glucose, HbA_1c_, fasting plasma insulin, and triglycerides levels. They were also less physically active, more often smoker, more frequently used lipid-modifying, antihypertensive or glucose-lowering medication, and more often had a history of CVD, decreased eGFR, albuminuria, and retinopathy. Of note, IGP tertiles did not fully correspond with GMS. Several individuals with type 2 diabetes (n = 47; 17 newly diagnosed) were not in the highest IGP tertile. Individuals with prediabetes were distributed equally among the second and third tertile. Heterogeneity existed regarding the OGTT glucose peak time point (Additional file 1: Table S3); for the first, second and third IGP tertile, the most frequently occurring time points were 30, 45 and 90 min, respectively.

**Table 1:** Participant characteristics according to incremental glucose peak (IGP) tertiles

Characteristic	First tertile (n = 924)	Second tertile (n = 909)	Third tertile (n = 925)
Age, years	57.3 ± 8.2	60.0 ± 8.0	62.1 ± 7.7
Women	569 (61.6)	431 (47.4)	330 (35.7)
Body mass index, kg/m^2^	25.2 ± 3.6	26.4 ± 3.9	28.8 ± 4.6
Waist circumference, cm			
Men	95.1 ± 9.1	98.4 ± 10.0	105.2 ± 11.3
Women	85.4 ± 10.5	89.1 ± 11.4	96.5 ± 13.9
Office SBP, mmHg	129.3 ± 16.6	134.2 ± 17.3	140.2 ± 17.7
Office DBP, mmHg	74.7 ± 9.5	76.7 ± 10.3	78.1 ± 9.6
Ambulatory 24-h SBP, mmHg	116.6 ± 10.9	119.0 ± 10.9	121.9 ± 12.4
Ambulatory 24-h DBP, mmHg	73.6 ± 7.0	74.5 ± 7.2	74.3 ± 7.4
Mean arterial pressure, mmHg	94.3 ± 10.3	96.9 ± 10.4	98.8 ± 10.1
Carotid pulse pressure, mmHg	46.3 ± 14.1	49.7 ± 14.7	52.3 ± 16.0
Mean heart rate, beats/min	60.7 ± 8.1	61.8 ± 9.0	64.5 ± 10.0
Physical activity, hours/week	14.0 [9.5–19.0]	13.5 [8.3–19.0]	11.5 [7.5–17.4]
Mediterranean diet score, (range: 0–9)	4.6 ± 1.7	4.6 ± 1.7	4.3 ± 1.6
Smoking			
Never/former/current	368/451/91	315/463/125	261/511/135
Never/former/current, %	40.4/49.6/10.0	34.9/51.3/13.8	28.8/56.3/14.9
Fasting plasma glucose (FPG), mmol/L	5.2 ± 0.5	5.5 ± 0.7	6.8 ± 1.3
2-h post-load glucose, mmol/L	5.1 ± 1.1	6.3 ± 1.7	12.3 ± 4.6
Glucose metabolism status			
NGM/prediabetes/type 2 diabetes	858/53/13	672/203/34	132/192/601
NGM/prediabetes/type 2 diabetes, %	92.9/5.7/1.4	73.9/22.3/3.7	14.3/20.8/65.0
Newly diagnosed type 2 diabetes	10 (1.1)	7 (0.8)	92 (9.9)
Incremental glucose peak (IGP), mmol/L	2.2 [1.8–2.7]	4.1 [3.6–4.7]	8.1 [6.5–10.0]
HbA_1c_, %	5.4 ± 0.3	5.6 ± 0.4	6.3 ± 0.7
HbA_1c_, mmol/mol	35.8 ± 3.7	37.4 ± 4.5	45.2 ± 7.8
Fasting plasma insulin, pmol/L	52.4 [38.3–71.0]	59.6 [41.7–86.8]	81.9 [51.5–125.5]
HOMA2-IR	1.2 [0.9–1.5]	1.3 [1.0–2.0]	2.0 [1.2–2.9]
Triglycerides, mmol/L	1.0 [0.8–1.4]	1.2 [0.9–1.6]	1.5 [1.1–2.1]
Total-to-HDL cholesterol ratio	3.3 [2.8–4.1]	3.5 [2.9–4.4]	3.6 [3.0–4.5]
Total cholesterol, mmol/L	5.5 ± 1.0	5.5 ± 1.1	4.9 ± 1.2
LDL cholesterol, mmol/L	3.4 ± 0.9	3.3 ± 1.0	2.8 ± 1.1
HDL cholesterol, mmol/L	1.7 ± 0.4	1.6 ± 0.5	1.3 ± 0.4
Lipid-modifying medication use	145 (15.7)	219 (24.1)	558 (60.5)
Antihypertensive medication use	197 (21.3)	276 (30.4)	546 (59.2)
Diabetes medication use	1 (0.1)	21 (2.3)	449 (48.6)
Insulin	0 (0)	0 (0)	0 (0)
Metformin	1 (0.1)	20 (2.2)	423 (45.8)
Sulfonylureas	0 (0)	3 (0.3)	149 (16.1)
Thiazolidinediones	0 (0)	1 (0.1)	8 (0.9)
GLP-1 analogs	0 (0)	0 (0)	6 (0.7)
DDP-4 inhibitors	0 (0)	1 (0.1)	50 (5.4)
History of CVD	110 (12.3)	116 (13.1)	174 (19.4)
eGFR, mL/min/1.73 m^2^	90.7 ± 13.2	88.5 ± 13.3	86.0 ± 15.6
eGFR < 60 mL/min/1.73 m^2^	14 (1.5)	20 (2.2)	58 (6.3)
(Micro)albuminuria	31 (3.4)	49 (5.4)	120 (13.0)
Retinopathy	0 (0)	2 (0.2)	14 (1.6)
Carotid-femoral pulse wave velocity (cf-PWV), m/s	8.3 ± 1.7	8.8 ± 1.9	9.7 ± 2.2
Carotid distensibility coefficient (carDC), 10^−3^/kPa	15.6 ± 5.4	14.4 ± 5.1	13.2 ± 4.8
Carotid intima-media thickness (cIMT), µm	846.3 ± 150.3	854.4 ± 155.8	876.1 ± 161.1
Mean circumferential wall stress (CWS_mean_), kPa	43.8 [37.8–50.9]	46.5 [40.8–53.0]	47.5 [41.0–56.0]
Pulsatile circumferential wall stress (CWS_puls_), kPa	20.9 [16.3–26.4]	23.1 [18.6–28.9]	24.2 [19.0–31.3]
Retinal arteriolar average dilatation, %	3.1 [1.1–5.3]	2.8 [1.1–5.2]	2.1 [0.5–4.4]
Heat-induced skin hyperemia, %	1110.5 [666.3–1592.3]	1027.6 [633.3–1587.3]	868.6 [521.3–1318.0]

Data are reported as mean ± SD, median [interquartile range], or number (percentage %) as appropriate. Data represent the study population of participants with complete oral glucose tolerance test data and results of at least one primary outcome

*CVD* cardiovascular disease, *SBP* systolic blood pressure, *DBP* diastolic blood pressure, *NGM* normal glucose metabolism, *HbA*_*1c*_ glycated hemoglobin A_1c_, *HOMA2-IR* updated homeostasis model assessment, *HDL* high-density lipoprotein, *LDL* low-density lipoprotein, *GLP-1* glucagon-like peptide-1, *DPP-4* dipeptidase-4, *eGFR* estimated glomerular filtration rate

### Incremental glucose peak and arterial stiffness

**Fig. 2:**
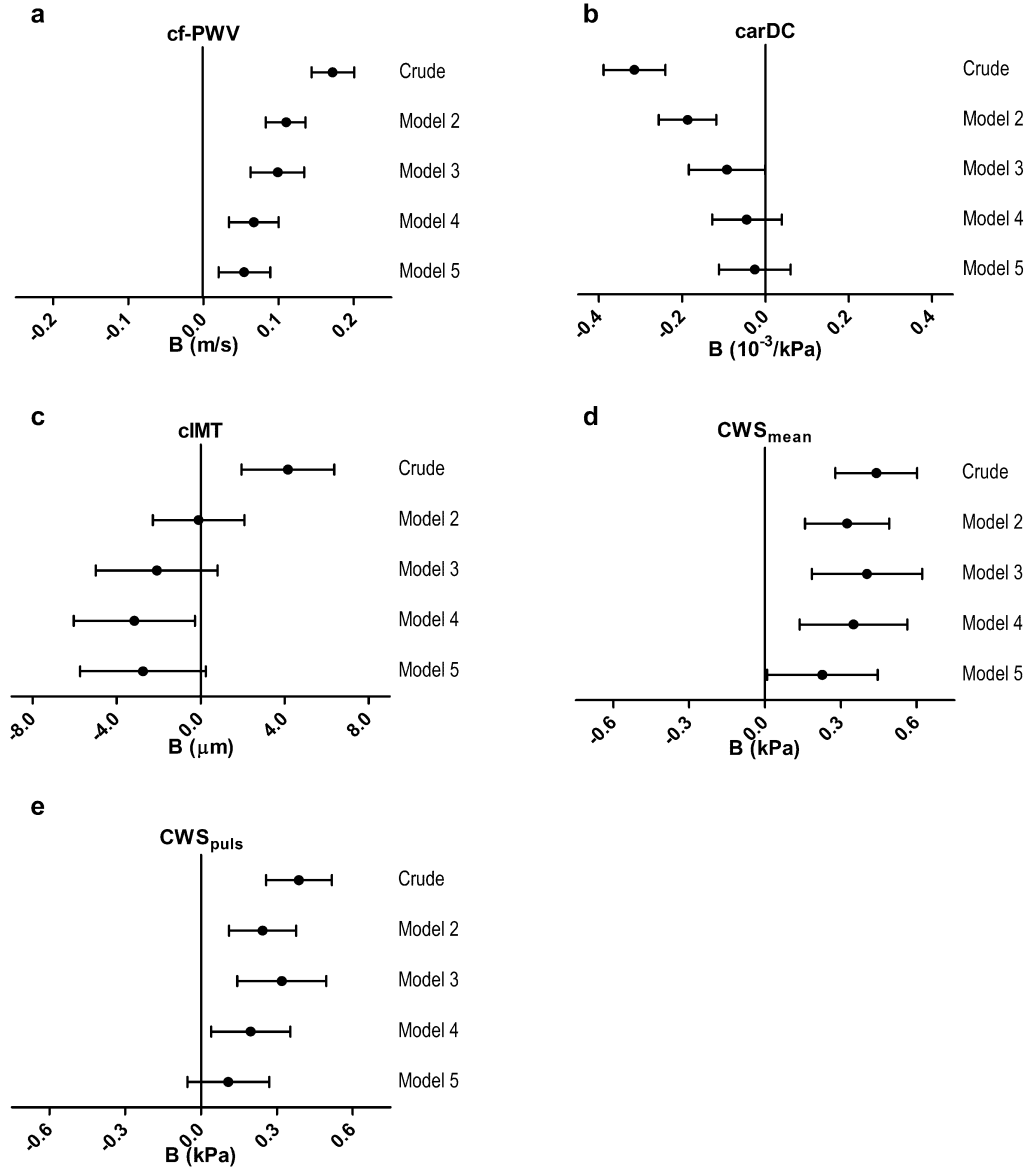
Multivariable-adjusted associations of incremental glucose peak (IGP) and arterial stiffness and arterial remodeling. Regression coefficients (B) indicate the mean difference (95% confidence interval) associated with 1 unit (mmol/L) increase of IGP. The panels depict the **a** associations between IGP and carotid-femoral pulse wave velocity (cf-PWV); **b** associations between IGP and carotid distensibility coefficient (carDC); **c** associations between IGP and carotid intima-media thickness (cIMT); **d** associations between IGP and mean circumferential wall stress (CWS_mean_); **e** associations between IGP and pulsatile circumferential wall stress (CWS_puls_). Model 1: crude. Model 2: additionally adjusted for age and sex. Model 3: additionally adjusted for HbA_1c_. Model 4: additionally adjusted for mean arterial pressure and mean heart rate (cf-PWV), mean arterial pressure (carDC, CWS_puls_), office systolic blood pressure (cIMT) or carotid pulse pressure (CWS_mean_). Model 5: additionally adjusted for body mass index, smoking status, physical activity, Mediterranean diet score, use of antihypertensive and lipid-modifying drugs, fasting triglycerides, and total-to-HDL cholesterol levels

Figure 2a, b and Additional file 1: Table S4 show the associations of IGP with cf-PWV and carDC. Higher IGP was statistically significantly associated with higher cf-PWV in the crude analysis. This association persisted after adjustment for age, sex, and HbA_1c_ (model 3), and additional adjustment for MAP and HR (model 4). After further adjustment for cardiovascular risk and lifestyle factors, the association of IGP with cf-PWV remained statistically significant (model 5, B: 0.054 m/s [0.020; 0.089], P = 0.002).

Higher IGP was statistically significantly associated with lower carDC in the crude analysis. This association persisted after adjustment for age, sex, and HbA_1c_ (model 3). The association did not remain statistically significant after adjustment for MAP, and cardiovascular risk factors and lifestyle factors (model 5, B: − 0.026 10^−3^/kPa [− 0.112; 0.060], P = 0.551).

### Incremental glucose peak, intima-media thickness and circumferential wall stress

Figure 2c–e and Additional file 1: Table S4 show the associations of IGP with cIMT, CWS_mean_ and CWS_puls_. There was a statistically significant, positive association between IGP and cIMT in the crude analysis (crude, B: 4.157 µm [1.944; 6.370], P < 0.001). Of note, this association became negative after correction for age, sex, and HbA_1c_ (model 3). Conversely, HbA_1c_ was positively associated with cIMT (data not shown). Higher IGP was not statistically significantly associated with lower cIMT in the fully adjusted model (model 5, B: − 2.745 µm [− 5.736; 0.245], P = 0.072).

Higher IGP was associated with higher CWS_mean_ in the crude model, and remained associated after adjustment for age, sex, and HbA_1c_ (model 3). The association between IGP and CWS_mean_ remained statistically significant after further correction for cardiovascular risk factors and lifestyle factors (model 5, B: 0.227 kPa [0.008; 0.446], P = 0.043).

IGP was positively associated with CWS_puls_ in the crude model, and after additional adjustment for age, sex, and HbA_1c_ (model 3). The association between IGP and CWS_puls_ did not remain statistically significant after correction for cardiovascular risk and lifestyle factors (model 5, B: 0.108 kPa [− 0.054; 0.270], P = 0.192).

### Incremental glucose peak and microvascular function

IGP was not associated with retinal arteriolar baseline diameter or skin baseline blood flow (Table 2). Higher IGP was statistically significantly associated with lower retinal arteriolar average dilatation and lower heat-induced skin hyperemia (crude models, Table 2). These associations did not remain statistically significant after adjustment for age, sex, and HbA_1c_ (retinal arteriolar average dilatation), and age and sex (heat-induced skin hyperemia).

**Table 2:** Multivariable-adjusted associations of incremental glucose peak (IGP) and microvascular function

Model	B (95% CI)	*P* value
Retinal arteriolar baseline diameter, MU (n = 1591)		
Crude	− 0.035 (− 0.295; 0.225)	0.792
Model 2	0.068 (− 0.204; 0.339)	0.626
Model 3	− 0.145 (− 0.503; 0.213)	0.428
Model 4	− 0.092 (− 0.451; 0.267)	0.614
Model 5	− 0.157 (− 0.528; 0.214)	0.406
Retinal arteriolar average dilatation, % (n = 1591)		
Crude	− 0.088 (− 0.134; − 0.043)	< 0.001
Model 2	− 0.073 (− 0.121; − 0.026)	0.002
Model 3	− 0.038 (− 0.101; 0.024)	0.229
Model 4	− 0.042 (− 0.105; 0.020)	0.184
Model 5	− 0.022 (− 0.087; 0.043)	0.506
Skin baseline blood flow, PU (n = 1134)		
Crude	0.016 (− 0.110; 0.142)	0.799
Model 2	− 0.024 (− 0.155; 0.107)	0.722
Model 3	0.025 (− 0.149; 0.198)	0.780
Model 4	0.049 (− 0.126; 0.224)	0.581
Model 5	0.065 (− 0.117; 0.246)	0.485
Heat-induced skin hyperemia, % (n = 1134)		
Crude	− 28.109 (− 42.778; − 13.440)	< 0.001
Model 2	− 12.503 (-27.509; 2.504)	0.102
Model 3	− 3.311 (− 23.208; 16.586)	0.744
Model 4	− 5.332 (− 25.420; 14.756)	0.603
Model 5	− 1.380 (− 22.273; 19.513)	0.897

Regression coefficients (B) indicate the mean difference (95% confidence interval) associated with 1 unit (mmol/L) increase of IGP. Model 1: crude. Model 2: additionally adjusted for age and sex. Model 3: additionally adjusted for HbA_1c_. Model 4: additionally adjusted for office systolic blood pressure. Model 5: additionally adjusted for body mass index, smoking status, physical activity, Mediterranean diet score, use of antihypertensive and lipid-modifying drugs, fasting triglycerides, and total-to-HDL cholesterol levels

### Additional analyses

Additional adjustment for history of CVD, retinopathy, eGFR, and urinary albumin excretion did not materially alter the results (Additional file 1: Tables S5 and S6), although statistical significance was not retained in the associations of IGP with CWS_mean_ (model 6). Additional adjustment for fasting plasma insulin or HOMA2-IR did not materially affect the results (Additional file 1: Table S7). The GMS- or FPG-adjusted models yielded results that were mostly comparable with the main (i.e. HbA_1c_-adjusted) models (Additional file 1: Table S8). Differences from the main models with regard to statistical significance were observed for the GMS-adjusted association of IGP and cf-PWV (5a, B: 0.031 m/s [− 0.008; 0.071], P = 0.120), the GMS-adjusted association of IGP and cIMT (5a, B: − 4.282 µm [− 7.706; -0.857], P = 0.014), and the FPG-adjusted association of IGP and CWS_mean_ (5b, B: 0.156 kPa [− 0.065; 0.377], P = 0.167). In general, the results were not materially different when AGP or IGP_percentage_ were used as determinant instead of IGP (Additional file 1: Tables S9 and S10). Adjustment for alternative BP measurements did not materially affect the results either (Additional file 1: Tables S11, S12 and S13). When time to glucose peak was used as determinant instead of IGP, only cf-PWV was found to be statistically significantly associated (B: 0.005 m/s [0.001; 0.008], *P* value = 0.007) (Additional file 1: Table S14).

The association between IGP and cf-PWV was stronger with higher age (*P* value for interaction < 0.001; Additional file 1: Table S15). The association between IGP and carDC was weaker with higher age (*P* value for interaction < 0.001; Additional file 1: Table S16). Age statistically significantly modified the association between IGP and CWS_puls_ (*P* value for interaction = 0.013; Additional file 1: Table S17). Sex did not modify the associations of IGP with arterial stiffness, arterial remodeling, or microvascular function.

## Discussion

In the present study, we investigated cross-sectional associations of IGP with arterial stiffness, arterial remodeling and microvascular function. Our study has two main findings. First, higher IGP was independently associated with higher aortic stiffness (cf-PWV) and higher CWS_mean_, but not with carotid stiffness (carDC), cIMT and CWS_puls_. Second, IGP was not independently associated with measures of microvascular function.

Our study shows that IGP measured during an OGTT provides additional information on top of established glycemic indices (i.e. HbA_1c_, GMS, and FPG). We found that IGP not fully corresponds with GMS, as individuals with prediabetes were equally distributed among the second and third IGP tertile (Table 1). Furthermore, we showed that the associations of IGP with cf-PWV and CWS_mean_ were independent of HbA_1c_.

### Current observations in the perspective of prior research

This is the first study to report on the association of IGP with arterial stiffness measures. Our findings are in concordance with studies using comparable determinants or outcomes. Hulman et al. for example, showed that individuals with the highest glucose peak during an OGTT were characterized by a worse cardiometabolic risk factor profile (i.e. age, sex, smoking status, BP, plasma lipids) [30]. Moreover, the 1-h OGTT value has previously been found to be independently associated with cf-PWV [31] and brachial-ankle PWV [29]. Still, in our study IGP was observed at this time point in only 20.8% of the participants (Additional file 1: Table S3). Our independent association between time to glucose peak and arterial stiffness (Additional file 1: Table S14) is also in line with Hulman et al.’s findings on glucose peak time point and cardiometabolic risk [30]. By contrast, in a study by the same research group no independent association between OGTT glucose peak and incident CVD was found [32]. However, their use of just a three-point OGTT entails a major limitation.

We observed a negative, albeit not statistically significant, association of IGP with cIMT, which is in contrast with two studies that have found a positive association between IGP and cIMT [33, 34]. These associations, however, were not adjusted for HbA_1c_ or other glycaemic indices. Adjustment for HbA_1c_, GMS or FPG consistently resulted in a negative association between IGP and cIMT.

The absence of a HbA_1c_-independent association between IGP and measures of microvascular function is in line with current literature to the extent that the association of glucose variability, as assessed by continuous glucose monitoring, with macroalbuminuria disappeared after adjustment for mean sensor glucose [11]. This could imply that mean glucose values, rather than glucose peaks, are an important determinant of microvascular function.

### Mechanistic explanations

The biological mechanism underlying the relationship between IGP and aortic stiffness remains to be elucidated. Previous research has shown that the glucose peak during an OGTT correlates well with glucose variability based on a self-determined ten-point home glucose profile [17]. Greater daily glucose variability may lead to greater oxidative stress [14, 15], which in turn could lead to advanced glycation end product (AGE) formation [2]. AGEs are thought to induce arterial stiffening by accumulating in the arterial wall and forming cross-links between elastin and collagen [3, 35]. Of interest, a previous study by our group showed that AGE precursor levels peaked in parallel with glucose values during an OGTT [36]. This supports the mechanistic concept that the mean glucose (reflected by HbA_1c_) and glucose variability (reflected by IGP) both contribute to arterial stiffness, mediated by AGEs. Alternatively, elevated IGP could be a hallmark of higher IR, which may, just as hyperinsulinemia, cause arterial stiffening [3, 37]. Indeed, glucose peak height and time point were associated with higher indices of IR in our study, as recently reported by Wang et al. [38]. However, additional adjustment for fasting plasma insulin or HOMA-IR did not substantially alter the results (Additional file 1: Table S7).

### Reflections on unexpected findings

Our analyses yielded several interesting findings. First, IGP was independently associated with cf-PWV, but not with carDC. This difference could be due to structural differences between the aorta (mixed elastic and muscular) and carotid artery (predominately elastic) [22]. Indeed, while an association of tissue and circulating AGEs with cf-PWV has been reported [39], no link has been established for carDC [40]. Second, the inverse association between IGP and cIMT was unexpected, in particular because we observed a positive association of cIMT with HbA_1c_, and found a statistically significant positive association between IGP and CWS_mean_, which normally should stimulate arterial remodeling to increase arterial wall thickness [25]. Our findings could therefore imply that individuals with high IGP values experience maladaptive arterial remodeling of the carotid artery, as has previously been demonstrated in patients with type 2 diabetes [25]. Alternatively, although the assumptions of linear regression were met and the sensitivity analyses showed comparable results, our findings could still be spurious (type 1 error). Third, IGP was statistically significantly associated with CWS_mean_, but not with CWS_puls_. Based on this finding and on the notion that the regression coefficients decreased more from model 3 to 4 for CWS_puls_ compared to CWS_mean_, we conclude that IGP is more strongly associated with MAP than with carPP, and thus that in our study population IGP corresponds more with mean than pulsatile vascular stress. Fourth, while we observed an independent association of IGP with the macrovasculature, no such association was found with the microvasculature, which might be attributable to sample size differences. Future research should focus on further elucidating this discrepancy. Fifth, the regression coefficients of the HbA_1c_-adjusted models differed in magnitude from the GMS- and FPG-adjusted models, which were performed as sensitivity analyses. This could be a result of using a categorical (i.e. GMS) instead of a continuous (i.e. HbA_1c_ and FPG) confounder [41]. Still, these associations were not statistically significantly dissimilar, as the 95% CIs strongly overlap and include each other’s regression coefficients [42]. The loss of statistical significance after adjustment for additional variables (e.g. history of CVD, eGFR) could be due to smaller sample size or overadjustment bias [43].

### Clinical relevance

Aortic stiffness, as measured by cf-PWV, is an independent determinant of CVD, cardiovascular mortality, and all-cause mortality [22]. We found that after adjustment for HbA_1c_ and all other relevant confounders cf-PWV was 0.054 m/s higher per IGP unit (mmol/L). This corresponds with six months of vascular aging per 1 mmol/L higher IGP [44]. Accordingly, the 5.9 mmol/L difference in median IGP between the first and third IGP tertile reflects a three year vascular aging difference. Our results may imply that, even in case of well-controlled HbA_1c_, the harmful effects of glucose peaks on aortic stiffness are still present. Future studies should investigate whether these findings translate to daily glucose fluctuations. If they are replicated using continuous glucose monitoring data, it would further justify therapeutic interventions that specifically target glucose variability.

### Strengths and limitations

This study has several strengths and limitations. Strengths are (1) the use of multiple, state-of-the-art measurements to study arterial stiffness, arterial remodeling, and microvascular function; (2) the study sample size and the extensive participant characterization, allowing adjustment for a broad array of possible confounders; and (3) the robustness of the results, reflected by the consistency of several sensitivity analyses. Our study had certain limitations. First, we could only calculate the main determinant using one OGTT, which is known for its moderate reproducibility [45]. The consequent random measurement error in IGP may have resulted in underestimated associations (i.e. attenuation bias) [46]. Second, a relatively large number of individuals were excluded due to missing determinant, outcome and/or confounder data (Fig. 1). Still, individuals with missing data were generally comparable to the final study population (Additional file 1: Table S1), except for the participants with an OGTT contraindication, who were characterized by a more adverse cardiometabolic profile. The inability to calculate IGP in this relatively unhealthy subgroup might have affected the precision of the associations. Third, the cross-sectional design renders us unable to rule out reverse causality. Arterial stiffness, which has been associated with incident diabetes, could theoretically influence glucose values [47]. Fourth, our study population is mostly Caucasian, which limits the generalizability of our results. Fifth, although the models were adjusted for a large number of cardiovascular risk and lifestyle factors, residual confounding may still be present.

## Conclusions

We show that higher IGP is independently associated with greater aortic stiffness and maladaptive carotid remodeling, but not with carotid stiffness, cIMT, or microvascular function. Taken together, these findings support the concept that glucose peaks have harmful macrovascular effects, regardless of mean glucose levels. Further research is needed to elucidate how these findings translate to daily glucose fluctuations and to what extent CVD could be prevented by reducing glucose variability.

## Supplementary information

**Additional file 1.** Additional tables.

## Data Availability

Data are available from The Maastricht Study for any researcher who meets the criteria for access to confidential data; the corresponding author may be contacted to request data.

## Abbreviations

AGE: advanced glycation end product
AGP: absolute glucose peak (during an OGTT)
B: regression coefficient
BMI: body mass index
BP: blood pressure
braPP: brachial pulse pressure
carDC: carotid distensibility coefficient
carPP: carotid pulse pressure
cf-PWV: carotid-femoral pulse wave velocity
CI: confidence interval
cIMT: carotid intima-media thickness
CVD: cardiovascular disease
CWS_mean_: mean circumferential wall stress
CWS_puls_: pulsatile circumferential wall stress
DVA: Dynamic Vessel Analyzer
eGFR: estimated glomerular filtration rate
FPG: fasting plasma glucose
GMS: glucose metabolism status
HOMA2-IR: updated Homeostatic Model Assessment
HR: heart rate
IAD: interadventitial diameter
IGP: incremental glucose peak (during an OGTT)
IGP_percentage_: percentage increase from baseline (during an OGTT)
IQR: interquartile range
IR: insulin resistance
LD: lumen diameter
MAP: mean arterial pressure
MU: measurement unit
NGM: normal glucose metabolism
OGTT: oral glucose tolerance test
PU: perfusion unit
SD: standard deviation
