# Supplementary material for: The oral glucose tolerance test-derived incremental glucose peak is associated with greater arterial stiffness and maladaptive arterial remodeling: The Maastricht Study
Source: Cardiovasc Diabetol. 2019 Nov 14;18:152. doi: 10.1186/s12933-019-0950-x (PMC6857146; doi:10.1186/s12933-019-0950-x)
Supplement: Supplementary file 1 — Additional file 1. Additional tables. [file 12933_2019_950_MOESM1_ESM.docx]

**Additional file to ‘The Oral Glucose Tolerance Test-Derived Incremental Glucose Peak Is Associated With Greater Arterial Stiffness And Maladaptive Arterial Remodeling: The Maastricht Study’**

**Additional methods**

*Statistical analysis*

We compared the groups stratified according to missing data reason with the total study population using independent T test in case of normally distributed continuous variables, Mann-Whitney U test in case of non-normally distributed continuous variables, and chi-square analysis in case of categorical variables.

**Additional results**

**Additional file 1: Table S1. Participant characteristics of the final study population and individuals excluded from the analyses due to missing data**

| **Characteristic** | **IGP study population (n=2758)** | **Missing OGTT data, OGTT contraindication (n=238)** | ***P* value*** | **Missing OGTT data,**  **missing samples (n=368)** | ***P* value*** | **Missing all outcome**  **data (n=46)** | ***P* value*** |
| --- | --- | --- | --- | --- | --- | --- | --- |
| Age, y | 59.8±8.2 | 62.0±8.0 | < 0.001 | 58.4±8.6 | 0.002 | 59.4±8.9 | NS |
| Women | 1,330 (48.2) | 65 (27.3) | < 0.001 | 238 (64.7) | < 0.001 | 21 (45.7) | NS |
| Body mass index, kg/m^2^ | 26.8±4.3 | 31.1±5.3 | < 0.001 | 26.7±4.7 | < 0.001 | 27.4±5.0 | NS |
| Waist circumference, cm |  |  |  |  |  |  |  |
| Men | 100.4±11.2 | 112.6±14.2 | < 0.001 | 99.6±11.4 | NS | 103.2±14.7 | NS |
| Women | 89.4±12.5 | 104.3±14.8 | < 0.001 | 90.1±13.1 | NS | 90.2±12.2 | NS |
| Office SBP, mmHg | 134.6±17.8 | 145.3±19.4 | < 0.001 | 132.4±18.3 | 0.031 | 133.1±17.0 | NS |
| Office DBP, mmHg | 76.5±9.9 | 75.1±9.6 | 0.033 | 74.9±9.4 | 0.005 | 75.6±9.4 | NS |
| Ambulatory 24-hour SBP, mmHg | 119.1±11.6 | 125.3±12.7 | < 0.001 | 116.4±10.9 | < 0.001 | 118.0±12.9 | NS |
| Ambulatory 24-hour DBP, mmHg | 74.1±7.2 | 72.9±7.0 | 0.018 | 73.1±6.8 | 0.005 | 73.6±7.6 | NS |
| Mean arterial pressure, mmHg | 96.7±10.4 | 97.8±9.1 | NS | 96.3±10.2 | NS | N/A (n=1) | N/A |
| Carotid pulse pressure, mmHg | 49.4±15.1 | 56.3±15.6 | < 0.001 | 48.5±15.4 | NS | N/A (n=2) | N/A |
| Mean heart rate, beats/minute | 62.3±9.2 | 68.4±10.1 | < 0.001 | 62.3±9.0 | NS | N/A (n=1) | N/A |
| Physical activity, hours/week | 13.0 [8.3-18.5] | 10.3 [5.8-15.9] | < 0.001 | 14.3 [8.5-21.1] | 0.052 | 13.8 [9.3-18.0] | NS |
| Mediterranean diet score, (range: 0-9) | 4.5±1.7 | 3.8±1.5 | < 0.001 | 4.5±1.6 | NS | 4.4±1.7 | NS |
| Smoking |  |  |  |  |  |  |  |
| Never/former/current | 944/1,425/351 | 54/123/48 | < 0.001 | 146/163/51 | 0.037 | 16/18/10 | NS |
| Never/former/current, % | 34.7/52.4/12.9 | 24.0/54.7/21.3 |  | 40.6/45.3/14.2 |  | 36.4/40.9/22.7 |  |
| Fasting plasma glucose, mmol/L | 5.8±1.2 | 9.4±3.1 | < 0.001 | 5.7±1.2 | 0.087 | 5.7±1.0 | NS |
| 2-hour post-load glucose, mmol/L | 7.9±4.3 | N/A | N/A | 7.7±3.9 | NS | 7.2±4.1 | NS |
| Glucose metabolism status |  |  |  |  |  |  |  |
| NGM/prediabetes/type 2 diabetes | 1,662/448/648 | 0/0/238 | < 0.001 | 229/57/82 | NS | 33/6/7 | NS |
| NGM/prediabetes/type 2 diabetes , % | 60.3/16.2/23.5 | 0/0/100 |  | 62.2/15.5/22.3 |  | 71.7/13.0/15.2 |  |
| Newly diagnosed type 2 diabetes | 109 (4.0) | 1 (0.4) | < 0.001 | 22 (6.0) | 0.081 | 1 (2.2) | NS |
| Incremental glucose peak (IGP), mmol/L | 4.1 [2.7-6.5] | N/A | N/A | N/A | N/A | 3.2 [2.4-5.6] | 0.072 |
| HbA_1c_, % | 5.8±0.6 | 8.0±1.4 | < 0.001 | 5.7±0.6 | NS | 5.7±0.7 | NS |
| HbA_1c_, mmol/mol | 39.5±7.0 | 63.7±14.9 |  | 38.9±6.9 |  | 38.4±7.2 |  |
| Fasting plasma insulin, pmol/L | 61.0 [42.8-93.0] | 67.6 [39.4-116.0] | NS | 59.2 [40.7-83.3] | NS | 61.6 [41.8-114.0] | NS |
| HOMA2-IR | 1.4 [1.0-2.1] | 1.9 [1.2-2.8] | < 0.001 | 1.4 [0.9-1.9] | NS | 1.5 [1.0-2.6] | NS |
| Triglycerides, mmol/L | 1.2 [0.9-1.7] | 1.6 [1.1-2.3] | < 0.001 | 1.2 [0.9-1.7] | NS | 1.2 [1.0-1.7] | NS |
| Total-to-HDL cholesterol ratio | 3.5 [2.9-4.3] | 3.5 [2.7-4.4] | NS | 3.4 [2.8-4.3] | NS | 3.2 [2.7-4.0] | NS |
| Total cholesterol, mmol/L | 5.3±1.1 | 4.1±0.9 | < 0.001 | 5.4±1.1 | 0.020 | 5.1±1.0 | NS |
| LDL cholesterol, mmol/L | 3.1±1.0 | 2.1±0.7 | < 0.001 | 3.2±1.0 | NS | 3.0±0.9 | NS |
| HDL cholesterol, mmol/L | 1.5±0.5 | 1.2±0.4 | < 0.001 | 1.6±0.5 | 0.003 | 1.6±0.4 | NS |
| Lipid-modifying medication use | 922 (33.4) | 204 (85.7) | < 0.001 | 90 (24.5) | 0.001 | 14 (30.4) | NS |
| Antihypertensive medication use | 1,019 (36.9) | 195 (81.9) | < 0.001 | 122 (33.2) | NS | 19 (41.3) | NS |
| Diabetes medication use | 471 (17.1) | 235 (98.7) | < 0.001 | 54 (14.7) | NS | 5 (10.9) | NS |
| Insulin | 0 (0) | 216 (90.8) | < 0.001 | 0 (0) | N/A | 0 (0) | NS |
| Metformin | 444 (16.1) | 180 (75.6) | < 0.001 | 49 (13.3) | NS | 5 (10.9) | NS |
| Sulfonylureas | 152 (5.5) | 26 (10.9) | 0.002 | 17 (4.6) | NS | 3 (6.5) | NS |
| Thiazolidinediones | 9 (0.3) | 3 (1.3) | 0.064 | 1 (0.3) | NS | 0 (0) | NS |
| GLP-1 analogs | 6 (0.2) | 2 (0.9) | NS | 1 (0.3) | NS | 0 (0) | NS |
| DDP-4 inhibitors | 51 (1.9) | 4 (1.7) | NS | 4 (1.1) | NS | 0 (0) | NS |
| History of CVD | 400 (14.9) | 86 (38.7) | < 0.001 | 60 (16.7) | NS | 6 (13.6) | NS |
| eGFR, mL/min/1.73 m^2^ | 88.4±14.2 | 83.6±20.0 | 0.013 | 86.4±17.6 | NS | 90.6±11.2 | NS |
| eGFR < 60 mL/min/1.73 m^2^ | 92 (3.3) | 34 (14.6) | < 0.001 | 16 (4.6) | NS | 1 (2.2) | NS |
| (Micro)albuminuria | 200 (7.3) | 70 (30.6) | < 0.001 | 18 (5.0) | NS | 3 (7.3) | NS |
| Retinopathy | 16 (0.6) | 27 (12.7) | < 0.001 | 3 (0.9) | NS | 0 (0) | NS |
| Carotid-femoral pulse wave velocity (cf-PWV), m/s | 8.9±2.0 | 10.7±2.7 | < 0.001 | 8.7±2.1 | NS | N/A | N/A |
| Carotid distensibility coefficient (carDC), 10^-3^/kPa | 14.4±5.2 | 13.1±4.5 | < 0.001 | 14.1±5.0 | NS | N/A | N/A |
| Carotid intima-media thickness (cIMT), µm | 858.9±156.2 | 883.2±175.0 | 0.039 | 842.5±156.1 | 0.083 | N/A | N/A |
| Mean circumferential wall stress (CWS_mean_), kPa | 45.9 [39.7-53.4] | 49.4 [41.5-55.9] | < 0.001 | 45.3 [38.9-52.8] | NS | N/A | N/A |
| Pulsatile circumferential wall stress (CWS_puls_), kPa | 22.8 [17.8-28.9] | 28.1 [21.5-34.7] | <0.001 | 21.7 [17.5-27.3] | NS | N/A | N/A |
| Retinal arteriolar average dilatation (DVA), % | 2.7 [0.9-5.1] | 1.2 [0.05-2.9] | < 0.001 | 2.9 [1.3-4.9] | NS | N/A | N/A |
| Heat-induced skin hyperemia, % | 999.1 [588.9-1,512.9] | 823.8 [464.5-1,191.3] | < 0.001 | 1,147.0 [643.5-1,690.4] | NS | N/A | N/A |

Data are reported as mean ± SD, median [interquartile range], or number (percentage %) as appropriate. Data represent the study population of participants with complete oral glucose tolerance test data and results of at least one primary outcome. CVD, cardiovascular disease; SBP, systolic blood pressure; DBP, diastolic blood pressure; NGM, normal glucose metabolism; HbA_1c_, glycated hemoglobin A_1c_; HDL, high-density lipoprotein; LDL, low-density lipoprotein; GLP-1, glucagon-like peptide-1; DPP-4, dipeptidase-4; eGFR, estimated glomerular filtration rate.* *P* values > 0.10 are presented as non-significant (NS) for the sake of clarity.

| **Characteristic** | **IGP study population (n=2,758)** | **Missing data** | **Excluded due to missing OGTT and outcome values (n=652)** | **Missing data** |
| --- | --- | --- | --- | --- |
| Age, y | 59.8±8.2 | 0 | 59.8±8.6 | 0 |
| Women | 1,330 (48.2) | 0 | 324 (49.7) | 0 |
| Body mass index, kg/m^2^ | 26.8±4.3 | 2 | 28.3±5.4 | 1 |
| Waist circumference, cm |  | 3 |  | 1 |
| Men | 100.4±11.2 |  | 106.7±14.6 |  |
| Women | 89.4±12.5 |  | 93.0±14.5 |  |
| Office SBP, mmHg | 134.6±17.8 | 6 | 137.2±19.6 | 0 |
| Office DBP, mmHg | 76.5±9.9 | 2 | 75.0±9.5 | 0 |
| Ambulatory 24-hour SBP, mmHg | 119.1±11.6 | 323 | 119.7±12.4 | 93 |
| Ambulatory 24-hour DBP, mmHg | 74.1±7.2 | 323 | 73.0±6.9 | 93 |
| Mean arterial pressure, mmHg | 96.7±10.4 | 327 | 96.9±9.8 | 124 |
| Carotid pulse pressure, mmHg | 49.4±15.1 | 488 | 51.8±15.9 | 177 |
| Mean heart rate, beats/minute | 62.3±9.2 | 326 | 64.7±9.9 | 124 |
| Physical activity, hours/week | 13.0 [8.3-18.5] | 328 | 12.5 [7.5-18.8] | 120 |
| Mediterranean diet score, (range: 0-9) | 4.5±1.7 | 165 | 4.3±1.6 | 57 |
| Smoking |  | 38 |  | 23 |
| Never/former/current | 944/1,425/351 |  | 216/304/109 |  |
| Never/former/current, % | 34.7/52.4/12.9 |  | 34.3/48.3/17.3 |  |
| Fasting plasma glucose, mmol/L | 5.8±1.2 | 0 | 7.0±2.8 | 4 |
| 2-hour post-load glucose, mmol/L | 7.9±4.3 | 0 | 7.7±3.9 | 260 |
| Glucose metabolism status |  | 0 |  | 0 |
| NGM/prediabetes/type 2 diabetes | 1,662/448/648 |  | 262/63/327 |  |
| NGM/prediabetes/type 2 diabetes , % | 60.3/16.2/23.5 |  | 40.2/9.7/50.2 |  |
| Incremental glucose peak (IGP), mmol/L | 4.1 [2.7-6.5] | 0 | 3.2 [2.4-5.6] | 606 |
| Newly diagnosed type 2 diabetes | 109 (4.0) | 0 | 24 (3.7) | 0 |
| HbA_1c_, % | 5.8±0.6 | 1 | 6.5±1.4 | 12 |
| HbA_1c_, mmol/mol | 39.5±7.0 |  | 48.1±16.0 |  |
| Fasting plasma insulin, pmol/L | 61.0 [42.8-93.0] | 7 | 61.4 [40.5-98.2] | 41 |
| HOMA2-IR | 1.4 [1.0-2.1] | 105 | 1.5 [1.0-2.3] | 93 |
| Triglycerides, mmol/L | 1.2 [0.9-1.7] | 0 | 1.3 [0.9-1.9] | 4 |
| Total-to-HDL cholesterol ratio | 3.5 [2.9-4.3] | 0 | 3.4 [2.8-4.3] | 4 |
| Total cholesterol, mmol/L | 5.3±1.1 | 0 | 4.9±1.2 | 4 |
| LDL cholesterol, mmol/L | 3.1±1.0 | 0 | 2.8±1.0 | 4 |
| HDL cholesterol, mmol/L | 1.5±0.5 | 0 | 1.5±0.5 | 4 |
| Lipid-modifying medication use | 922 (33.4) | 4 | 308 (47.2) | 0 |
| Antihypertensive medication use | 1,019 (36.9) | 4 | 336 (51.5) | 0 |
| Diabetes medication use | 471 (17.1) | 4 | 294 (45.1) | 0 |
| Insulin | 0 (0) |  | 216 (33.1) |  |
| Metformin | 444 (16.1) |  | 234 (35.9) |  |
| Sulfonylureas | 152 (5.5) |  | 46 (7.1) |  |
| Thiazolidinediones | 9 (0.3) |  | 4 (0.6) |  |
| GLP-1 analogs | 6 (0.2) |  | 3 (0.5) |  |
| DDP-4 inhibitors | 51 (1.9) |  | 8 (1.2) |  |
| History of CVD | 400 (14.9) | 78 | 152 (24.3) | 27 |
| eGFR, mL/min/1.73 m^2^ | 88.4±14.2 | 6 | 86.8±17.3 | 27 |
| eGFR < 60 mL/min/1.73 m^2^ | 92 (3.3) |  | 51 (8.2) |  |
| (Micro)albuminuria | 200 (7.3) | 20 | 91 (14.4) | 22 |
| Retinopathy | 16 (0.6) | 266 | 30 (4.6) | 86 |
| Carotid-femoral pulse wave velocity (cf-PWV), m/s | 8.9±2.0 | 397 | 9.5±2.5 | 162 |
| Carotid distensibility coefficient (carDC), 10^-3^/kPa | 14.4±5.2 | 368 | 13.7±4.8 | 148 |
| Carotid intima-media thickness (cIMT), µm | 858.9±156.2 | 373 | 858.2±164.7 | 149 |
| Mean circumferential wall stress (CWS_mean_), kPa | 45.9 [39.7-53.4] | 377 | 46.4 [40.5-54.2] | 149 |
| Pulsatile circumferential wall stress (CWS_puls_), kPa | 22.8 [17.8-28.9] | 536 | 23.8 [19.1-30.1] | 193 |
| Retinal arteriolar average dilatation (DVA), % | 2.7 [0.9-5.1] | 877 | 2.3 [0.7-4.1] | 272 |
| Heat-induced skin hyperemia, % | 999.1 [588.9-1,512.9] | 1388 | 954.1 [560.6-1,387.4] | 375 |

**Additional file 1: Table S2. Participant characteristics of the final study population and individuals excluded from the analyses due to missing data**

Data are reported as mean ± SD, median [interquartile range], or number (percentage %) as appropriate. Data represent the study population of participants with complete oral glucose tolerance test data and results of at least one primary outcome. CVD, cardiovascular disease; SBP, systolic blood pressure; DBP, diastolic blood pressure; NGM, normal glucose metabolism; HbA_1c_, glycated hemoglobin A_1c_; HDL, high-density lipoprotein; LDL, low-density lipoprotein; GLP-1, glucagon-like peptide-1; DPP-4, dipeptidase-4; eGFR, estimated glomerular filtration rate.

**Additional file 1: Table S3. Glucose peak time point during oral glucose tolerance test for the total study population and incremental glucose peak (IGP) tertiles**

| **Peak time point during OGTT** | **Total study population**  **(n=2,758)** | **first tertile**  **(n=924)** | **second tertile**  **(n=909)** | **third tertile**  **(n=925)** |
| --- | --- | --- | --- | --- |
| Fasting plasma glucose (FPG) | 0 (0) | 0 (0) | 0 (0) | 0 (0) |
| 15 minutes | 151 (5.5) | 143 (15.5) | 8 (0.9) | 0 (0) |
| 30 minutes | 682 (24.7) | 436 (47.2) | 225 (24.8) | 21 (2.3) |
| 45 minutes | 758 (27.5) | 217 (23.5) | 407 (44.8) | 124 (14.5) |
| 60 minutes | 574 (20.8) | 76 (8.2) | 199 (21.9) | 299 (32.3) |
| 90 minutes | 446 (16.2) | 33 (3.6) | 50 (5.5) | 363 (39.2) |
| 120 minutes | 147 (5.3) | 19 (2.1) | 20 (2.2) | 108 (11.7) |

Data are reported as number (percentage %). OGTT, oral glucose tolerance test.

**Additional file 1: Table S4. Multivariable-adjusted associations of incremental glucose peak (IGP) and arterial stiffness and arterial remodeling**

| **Model** | **B (95%CI)** | ***P* value** |
| --- | --- | --- |
| *Carotid femoral pulse wave velocity (cf-PWV), m/s (n=1,954)* | | |
| Crude | 0.172 (0.144; 0.201) | < 0.001 |
| Model 2 | 0.110 (0.083; 0.136) | < 0.001 |
| Model 3 | 0.099 (0.063; 0.134) | < 0.001 |
| Model 4* | 0.067 (0.034; 0.100) | < 0.001 |
| Model 5 | 0.054 (0.020; 0.089) | 0.002 |
| *Carotid distensibility coefficient (carDC), 10^-3^/kPa (n=1,978)* | | |
| Crude | -0.315 (-0.389; -0.240) | < 0.001 |
| Model 2 | -0.187 (-0.256; -0.118) | < 0.001 |
| Model 3 | -0.093 (-0.184; -0.001) | 0.047 |
| Model 4* | -0.045 (-0.128; 0.039) | 0.294 |
| Model 5 | -0.026 (-0.112; 0.060) | 0.551 |
| *Carotid intima-media thickness (cIMT),* *µm (n=1,973)* | | |
| Crude | 4.157 (1.944; 6.370) | < 0.001 |
| Model 2 | -0.094 (-2.274; 2.087) | 0.933 |
| Model 3 | -2.089 (-4.980; 0.801) | 0.157 |
| Model 4† | -3.159 (-6.047; -0.271) | 0.032 |
| Model 5 | -2.745 (-5.736; 0.245) | 0.072 |
| *Mean circumferential wall stress (CWS_mean_), kPa (n=1,870)* | | |
| Crude | 0.441 (0.279; 0.603) | < 0.001 |
| Model 2 | 0.325 (0.159; 0.491) | < 0.001 |
| Model 3 | 0.404 (0.185; 0.623) | < 0.001 |
| Model 4‡ | 0.350 (0.137; 0.564) | 0.001 |
| Model 5 | 0.227 (0.008; 0.446) | 0.043 |
| *Pulsatile circumferential wall stress (CWS_puls_), kPa (n=1,849)* | | |
| Crude | 0.387 (0.257; 0.518) | < 0.001 |
| Model 2 | 0.244 (0.110; 0.377) | < 0.001 |
| Model 3 | 0.320 (0.144; 0.495) | < 0.001 |
| Model 4* | 0.196 (0.040; 0.353) | 0.014 |
| Model 5 | 0.108 (-0.054; 0.270) | 0.192 |

Regression coefficients (B) indicate the mean difference (95% confidence interval) associated with 1 unit (mmol/L) increase of IGP. Model 1: crude. Model 2: additionally adjusted for age and sex. Model 3: additionally adjusted for HbA_1c_. Model 4: additionally adjusted for mean arterial pressure (*), office systolic blood pressure (†) or carotid pulse pressure (‡) and heart rate in case of cf-PWV. Model 5: additionally adjusted for body mass index, smoking status, physical activity, Mediterranean diet score, use of antihypertensive and lipid-modifying drugs, fasting triglycerides, and total-to-HDL cholesterol levels.

**Additional file 1: Table S5. Multivariable-adjusted associations of incremental glucose peak (IGP) and arterial stiffness and arterial remodeling, additionally adjusted for history of cardiovascular disease, retinopathy, estimated glomerular filtration rate, and urinary albumin excretion**

| **Model** | **B (95%CI)** | **P-value** |
| --- | --- | --- |
| *Carotid femoral pulse wave velocity (cf-PWV), m/s (n=1,722)* | | |
| Crude | 0.168 (0.138; 0.199) | < 0.001 |
| Model 2 | 0.105 (0.076; 0.134) | < 0.001 |
| Model 3 | 0.096 (0.058; 0.134) | < 0.001 |
| Model 4* | 0.065 (0.030; 0.100) | < 0.001 |
| Model 5 | 0.056 (0.019; 0.093) | 0.003 |
| Model 6 | 0.054 (0.017; 0.091) | 0.004 |
| *Carotid distensibility coefficient (carDC), 10^-3^/kPa (n=1,744)* | | |
| Crude | -0.321 (-0.400; -0.242) | < 0.001 |
| Model 2 | -0.195 (-0.269; -0.121) | < 0.001 |
| Model 3 | -0.109 (-0.207; -0.011) | 0.029 |
| Model 4* | -0.060 (-0.149; 0.030) | 0.189 |
| Model 5 | -0.035 (-0.128; 0.057) | 0.453 |
| Model 6 | -0.031 (-0.124; 0.062) | 0.511 |
| *Carotid intima-media thickness (cIMT),* *µm (n=1,741)* | | |
| Crude | 4.045 (1.729; 6.362) | 0.001 |
| Model 2 | -0.197 (-2.489; 2.094) | 0.866 |
| Model 3 | -2.491 (-5.537; 0.555) | 0.109 |
| Model 4† | -3.415 (-6.463; -0.367) | 0.028 |
| Model 5 | -2.920 (-6.088; 0.248) | 0.071 |
| Model 6 | -2.827 (-6.016; 0.362) | 0.082 |
| *Mean circumferential wall stress (CWS_mean_), kPa (n=1,633)* | | |
| Crude | 0.408 (0.235; 0.582) | < 0.001 |
| Model 2 | 0.281 (0.102; 0.459) | 0.002 |
| Model 3 | 0.420 (0.184; 0.656) | < 0.001 |
| Model 4‡ | 0.372 (0.143; 0.601) | 0.001 |
| Model 5 | 0.250 (0.013; 0.486) | 0.039 |
| Model 6 | 0.224 (-0.013; 0.462) | 0.064 |
| *Pulsatile circumferential wall stress (CWS_puls_), kPa (n=1,633)* | | |
| Crude | 0.333 (0.194; 0.471) | < 0.001 |
| Model 2 | 0.188 (0.047; 0.329) | 0.009 |
| Model 3 | 0.280 (0.094; 0.466) | 0.003 |
| Model 4* | 0.152 (-0.014; 0.317) | 0.073 |
| Model 5 | 0.069 (-0.103; 0.242) | 0.431 |
| Model 6 | 0.066 (-0.108; 0.239) | 0.458 |

Regression coefficients (B) indicate the mean difference (95% confidence interval) associated with 1 unit (mmol/L) increase of IGP. Model 1: crude. Model 2: additionally adjusted for age and sex. Model 3: additionally adjusted for HbA_1c_. Model 4: additionally adjusted for mean arterial pressure (*), office systolic blood pressure (†) or carotid pulse pressure (‡) and heart rate in case of cf-PWV. Model 5: additionally adjusted for body mass index, smoking status, physical activity, Mediterranean diet score, use of antihypertensive and lipid-modifying drugs, fasting triglycerides, and total-to-HDL cholesterol levels. Model 6: additionally adjusted for history of cardiovascular disease, retinopathy, estimated glomerular filtration rate, and urinary albumin excretion.

**Additional file 1: Table S6. Multivariable-adjusted associations of incremental glucose peak (IGP) and microvascular function, additionally adjusted for history of cardiovascular disease, retinopathy, estimated glomerular filtration rate, and urinary albumin excretion**

| **Model** | **B (95%CI)** | | **P-value** |
| --- | --- | --- | --- |
| *Retinal arteriolar baseline diameter, MU (n=1,514)* | | | |
| Crude | -0.043 (-0.308; 0.223) | | 0.753 |
| Model 2 | 0.076 (-0.202; 0.354) | | 0.591 |
| Model 3 | -0.133 (-0.498; 0.232) | | 0.474 |
| Model 4 | -0.079 (-0.446; 0.287) | | 0.672 |
| Model 5 | -0.143 (-0.522; 0.235) | | 0.458 |
| Model 6 | -0.149 (-0.527; 0.230) | | 0.440 |
| *Retinal arteriolar average dilatation, % (n=1,514)* | | | |
| Crude | -0.091 (-0.138; -0.044) | | < 0.001 |
| Model 2 | -0.077 (-0.125; -0.028) | | 0.002 |
| Model 3 | -0.041 (-0.105; 0.022) | | 0.203 |
| Model 4 | -0.045 (-0.109; 0.019) | | 0.169 |
| Model 5 | -0.023 (-0.090; 0.043) | | 0.497 |
| Model 6 | -0.018 (-0.084; 0.049) | | 0.607 |
| *Skin baseline blood flow, PU (n=1,013)* | | | |
| Crude | 0.010 (-0.123; 0.143) | | 0.880 |
| Model 2 | -0.034 (-0.172; 0.105) | | 0.634 |
| Model 3 | 0.028 (-0.156; 0.211) | | 0.767 |
| Model 4 | 0.052 (-0.134; 0.237) | | 0.582 |
| Model 5 | 0.077 (-0.117; 0.270) | | 0.437 |
| Model 6 | 0.042 (-0.153; 0.237) | | 0.674 |
| *Heat-induced skin hyperemia, % (n=1,013)* | | | |
| Crude | -26.408 (-41.668; -11.147) | 0.001 | |
| Model 2 | -8.673 (-24.293; 6.946) | | 0.276 |
| Model 3 | -1.164 (-21.867; 19.540) | | 0.912 |
| Model 4 | -3.704 (-24.615; 17.208) | | 0.728 |
| Model 5 | -0.211 (-22.086; 21.664) | | 0.985 |
| Model 6 | 0.194 (-21.935; 22.322) | | 0.986 |

Regression coefficients (B) indicate the mean difference (95% confidence interval) associated with 1 unit (mmol/L) increase of IGP. Model 1: crude. Model 2: additionally adjusted for age and sex. Model 3: additionally adjusted for HbA_1c_. Model 4: additionally adjusted for office systolic blood pressure. Model 5: additionally adjusted for body mass index, smoking status, physical activity, Mediterranean diet score, use of antihypertensive and lipid-modifying drugs, fasting triglycerides, and total-to-HDL cholesterol levels. Model 6: additionally adjusted for history of cardiovascular disease, retinopathy, estimated glomerular filtration rate, and urinary albumin excretion.

**Additional file 1: Table S7. Multivariable-adjusted associations of incremental glucose peak (IGP) and arterial stiffness and arterial remodeling, additionally adjusted for fasting plasma insulin (6.1) or HOMA2-IR (6.2)**

| **Model** | **B (95%CI)** | **P-value** |
| --- | --- | --- |
| *Carotid femoral pulse wave velocity (cf-PWV), m/s (n=1,890)* | | |
| Crude | 0.174 (0.144; 0.203) | < 0.001 |
| Model 2 | 0.109 (0.081; 0.136) | < 0.001 |
| Model 3 | 0.101 (0.065; 0.138) | < 0.001 |
| Model 4* | 0.070 (0.036; 0.103) | < 0.001 |
| Model 5 | 0.056 (0.021; 0.091) | 0.002 |
| Model 6.1 | 0.056 (0.021; 0.091) | 0.002 |
| Model 6.2 | 0.056 (0.021; 0.091) | 0.002 |
| *Carotid distensibility coefficient (carDC), 10^-3^/kPa (n=1,908)* | | |
| Crude | -0.316 (-0.392; -0.240) | < 0.001 |
| Model 2 | -0.185 (-0.256; -0.114) | < 0.001 |
| Model 3 | -0.099 (-0.192; -0.005) | 0.039 |
| Model 4* | -0.050 (-0.135; 0.036) | 0.254 |
| Model 5 | -0.030 (-0.118; 0.059) | 0.509 |
| Model 6.1 | -0.030 (-0.118; 0.059) | 0.509 |
| Model 6.2 | -0.031 (-0.120; 0.058) | 0.491 |
| *Carotid intima-media thickness (cIMT),* *µm (n=1,903)* | | |
| Crude | 3.975 (1.710; 6.239) | 0.001 |
| Model 2 | -0.306 (-2.542; 1.929) | 0.788 |
| Model 3 | -2.105 (-5.064; 0.854) | 0.163 |
| Model 4† | -3.215 (-6.172; -0.258) | 0.033 |
| Model 5 | -2.791 (-5.859; 0.277) | 0.075 |
| Model 6.1 | -2.797 (-5.866; 0.272) | 0.074 |
| Model 6.2 | -2.782 (-5.851; 0.287) | 0.076 |
| *Mean circumferential wall stress (CWS_mean_), kPa (n=1,784)* | | |
| Crude | 0.445 (0.278; 0.612) | < 0.001 |
| Model 2 | 0.329 (0.157; 0.501) | < 0.001 |
| Model 3 | 0.406 (0.180; 0.633) | < 0.001 |
| Model 4‡ | 0.345 (0.124; 0.565) | 0.002 |
| Model 5 | 0.201 (-0.025; 0.428) | 0.082 |
| Model 6.1 | 0.202 (-0.025; 0.428) | 0.082 |
| Model 6.2 | 0.203 (-0.024; 0.430) | 0.079 |
| *Pulsatile circumferential wall stress (CWS_puls_), kPa (n=1,784)* | | |
| Crude | 0.394 (0.261; 0.527) | < 0.001 |
| Model 2 | 0.253 (0.117; 0.389) | < 0.001 |
| Model 3 | 0.326 (0.147; 0.505) | < 0.001 |
| Model 4* | 0.202 (0.043; 0.362) | 0.013 |
| Model 5 | 0.105 (-0.060; 0.271) | 0.213 |
| Model 6.1 | 0.105 (-0.060; 0.271) | 0.212 |
| Model 6.2 | 0.106 (-0.059; 0.272) | 0.209 |

Regression coefficients (B) indicate the mean difference (95% confidence interval) associated with 1 unit (mmol/L) increase of IGP. Model 1: crude. Model 2: additionally adjusted for age and sex. Model 3: additionally adjusted for HbA_1c_. Model 4: additionally adjusted for mean arterial pressure (*), office systolic blood pressure (†) or carotid pulse pressure (‡)and heart rate in case of cf-PWV. Model 5: additionally adjusted for body mass index, smoking status, physical activity, Mediterranean diet score, use of antihypertensive and lipid-modifying drugs, fasting triglycerides, and total-to-HDL cholesterol levels. Model 6 additionally adjusted for fasting plasma insulin (6.1) or HOMA2-IR (6.2).

**Additional file 1: Table S8. Multivariable-adjusted associations of incremental glucose peak (IGP) and arterial stiffness, arterial remodeling and microvascular function after adjustment for glucose metabolism status or fasting plasma glucose instead of HbA_1c_**

| **Model** | **B (95%CI)** | ***P* value** | **Model** | **B (95%CI)** | ***P* value** |
| --- | --- | --- | --- | --- | --- |
| *Carotid femoral pulse wave velocity (cf-PWV), m/s (n=1,954)* | | | | | |
| Crude | 0.172 (0.144; 0.201) | < 0.001 | Crude |  |  |
| Model 2 | 0.110 (0.083; 0.136) | < 0.001 | Model 2 |  |  |
| Model 3a | 0.074 (0.032; 0.116) | 0.001 | Model 3b | 0.079 (0.043; 0.114) | < 0.001 |
| Model 4a^*^ | 0.040 (0.001; 0.079) | 0.045 | Model 4b* | 0.061 (0.028; 0.094) | < 0.001 |
| Model 5a | 0.031 (-0.008; 0.071) | 0.120 | Model 5b | 0.048 (0.014; 0.082) | 0.006 |
| *Carotid distensibility coefficient (carDC), 10^-3^/kPa (n=1,978)* | | | | | |
| Crude | -0.315 (-0.389; -0.240) | < 0.001 | Crude |  |  |
| Model 2 | -0.187 (-0.256; -0.118) | < 0.001 | Model 2 |  |  |
| Model 3a | -0.162 (-0.270; -0.053) | 0.003 | Model 3b | -0.090 (-0.182; 0.002) | 0.054 |
| Model 4a^*^ | -0.109 (-0.208; -0.011) | 0.030 | Model 4b^*^ | -0.078 (-0.162; 0.005) | 0.067 |
| Model 5a | -0.095 (-0.194; 0.005) | 0.062 | Model 5b | -0.061 (-0.148; 0.026) | 0.166 |
| *Carotid intima-media thickness (cIMT),* *µm (n=1,973)* | | | | | |
| Crude | 4.157 (1.944; 6.370) | < 0.001 | Crude |  |  |
| Model 2 | -0.094 (-2.274; 2.087) | 0.933 | Model 2 |  |  |
| Model 3a | -3.582 (-6.991; -0.172) | 0.039 | Model 3b | -2.446 (-5.347; 0.454) | 0.098 |
| Model 4a^†^ | -4.181 (-7.568; -0.793) | 0.016 | Model 4b^†^ | -3.067 (-5.952; -0.182) | 0.037 |
| Model 5a | -4.282 (-7.706; -0.857) | 0.014 | Model 5b | -3.004 (-6.005; -0.002) | 0.050 |
| *Mean circumferential wall stress (CWS_mean_), kPa (n=1,870)* | | | | | |
| Crude | 0.441 (0.279; 0.603) | < 0.001 | Crude |  |  |
| Model 2 | 0.325 (0.159; 0.491) | < 0.001 | Model 2 |  |  |
| Model 3a | 0.429 (0.169; 0.689) | 0.001 | Model 3b | 0.258 (0.038; 0.479) | 0.022 |
| Model 4a^‡^ | 0.380 (0.127; 0.633) | 0.003 | Model 4b^‡^ | 0.219 (0.004; 0.434) | 0.046 |
| Model 5a | 0.343 (0.090; 0.596) | 0.008 | Model 5b | 0.156 (-0.065; 0.377) | 0.167 |
| *Pulsatile circumferential wall stress (CWS_puls_), kPa (n=1,849)* | | | | | |
| Crude | 0.387 (0.257; 0.518) | < 0.001 | Crude |  |  |
| Model 2 | 0.244 (0.110; 0.377) | < 0.001 | Model 2 |  |  |
| Model 3a | 0.265 (0.057; 0.472) | 0.013 | Model 3b | 0.214 (0.037; 0.391) | 0.018 |
| Model 4a^*^ | 0.126 (-0.059; 0.312) | 0.182 | Model 4b^*^ | 0.159 (0.001; 0.316) | 0.048 |
| Model 5a | 0.076 (-0.111; 0.264) | 0.423 | Model 5b | 0.067 (-0.097; 0.230) | 0.425 |
| *Retinal arteriolar average dilatation (n=1,591)* | | | | | |
| Crude | -0.088 (-0.134; -0.043) | < 0.001 | Crude |  |  |
| Model 2 | -0.073 (-0.121; -0.026) | 0.002 | Model 2 |  |  |
| Model 3a | 0.019 (-0.055; 0.093) | 0.619 | Model 3b | -0.026 (-0.089; 0.037) | 0.424 |
| Model 4a^†^ | 0.015 (-0.059; 0.090) | 0.689 | Model 4b^†^ | -0.029 (-0.092; 0.035) | 0.375 |
| Model 5a | 0.026 (-0.049; 0.101) | 0.501 | Model 5b | -0.006 (-0.072; 0.060) | 0.854 |
| *Heat-induced skin hyperemia (n=1,134)* | | | | | |
| Crude | -28.109 (-42.778; -13.440) | < 0.001 | Crude |  |  |
| Model 2 | -12.503 (-27.509; 2.504) | 0.102 | Model 2 |  |  |
| Model 3a | 1.347 (-22.597; 25.291) | 0.912 | Model 3b | -10.477 (-30.879; 9.926) | 0.314 |
| Model 4a^†^ | -0.043 (-24.042; 23.956) | 0.997 | Model 4b^†^ | -11.996 (-32.497; 8.505) | 0.251 |
| Model 5a | 4.340 (-19.989; 28.669) | 0.726 | Model 5b | -6.214 (-27.437; 15.009) | 0.566 |

Regression coefficients (B) indicate the mean difference (95% confidence interval) associated with 1 unit (mmol/L) increase of IGP. Model 1: crude. Model 2: additionally adjusted for age and sex. Model 3: additionally adjusted for glucose metabolism status (a, left column) or fasting plasma glucose (FPG) (b, right column). Model 4: additionally adjusted for mean arterial pressure (*), office systolic blood pressure (†) or carotid pulse pressure (‡) and heart rate in case of cf-PWV. Model 5: additionally adjusted for body mass index, smoking status, physical activity, Mediterranean diet score, use of antihypertensive and lipid-modifying drugs, fasting triglycerides, and total-to-HDL cholesterol levels.

**Additional file 1: Table S9. Multivariable-adjusted associations of absolute glucose peak (AGP) and arterial stiffness, arterial remodeling and microvascular function**

| **Model** | **B (95%CI)** | ***P* value** |
| --- | --- | --- |
| *Carotid femoral pulse wave velocity (cf-PWV), m/s (n=1,954)* | | |
| Crude | 0.144 (0.121; 0.166) | < 0.001 |
| Model 2 | 0.090 (0.069; 0.112) | < 0.001 |
| Model 3 | 0.092 (0.061; 0.123) | < 0.001 |
| Model 4^*^ | 0.059 (0.031; 0.088) | < 0.001 |
| Model 5 | 0.049 (0.019; 0.079) | 0.001 |
| *Carotid distensibility coefficient (carDC), 10^-3^/kPa (n=1,978)* | | |
| Crude | -0.268 (-0.326; -0.210) | < 0.001 |
| Model 2 | -0.163 (-0.217; -0.108) | < 0.001 |
| Model 3 | -0.097 (-0.176; -0.017) | 0.017 |
| Model 4^*^ | -0.042 (-0.115; 0.030) | 0.250 |
| Model 5 | -0.020 (-0.096; 0.055) | 0.598 |
| *Carotid intima-media thickness (cIMT),* *µm (n=1,973)* | | |
| Crude | 4.013 (2.290; 5.736) | < 0.001 |
| Model 2 | 0.380 (-1.338; 2.098) | 0.664 |
| Model 3 | -1.217 (-3.719; 1.285) | 0.340 |
| Model 4^†^ | -2.296 (-4.803; 0.212) | 0.073 |
| Model 5 | -1.778 (-4.389; 0.833) | 0.182 |
| *Mean circumferential wall stress (CWS_mean_), kPa (n=1,870)* | | |
| Crude | 0.363 (0.237; 0.490) | < 0.001 |
| Model 2 | 0.263 (0.132; 0.394) | < 0.001 |
| Model 3 | 0.382 (0.192; 0.572) | < 0.001 |
| Model 4^‡^ | 0.333 (0.148; 0.517) | < 0.001 |
| Model 5 | 0.200 (0.008; 0.391) | 0.041 |
| *Pulsatile circumferential wall stress (CWS_puls_), kPa (n=1,849)* | | |
| Crude | 0.315 (0.213; 0.417) | < 0.001 |
| Model 2 | 0.193 (0.088; 0.299) | < 0.001 |
| Model 3 | 0.296 (0.144; 0.447) | < 0.001 |
| Model 4^*^ | 0.167 (0.031; 0.302) | 0.016 |
| Model 5 | 0.087 (-0.054; 0.228) | 0.228 |
| *Retinal arteriolar average dilatation (n=1,591)* | | |
| Crude | -0.076 (-0.111; -0.041) | < 0.001 |
| Model 2 | -0.065 (-0.102; -0.028) | 0.001 |
| Model 3 | -0.043 (-0.097; 0.011) | 0.116 |
| Model 4^†^ | -0.048 (-0.102; 0.007) | 0.085 |
| Model 5 | -0.031 (-0.087; 0.026) | 0.284 |
| *Heat-induced skin hyperemia (n=1,134)* | | |
| Crude | -23.140(-34.467; -11.813) | < 0.001 |
| Model 2 | -9.906 (-21.625; 1.813) | 0.097 |
| Model 3 | -1.556 (-18.602; 15.490) | 0.858 |
| Model 4^†^ | -3.408 (-20.647; 13.831) | 0.698 |
| Model 5 | -0.757 (-18.811; 17.297) | 0.934 |

Regression coefficients (B) indicate the mean difference (95% confidence interval) associated with 1 unit (mmol/L) increase of absolute glucose peak (AGP). Model 1: crude. Model 2: additionally adjusted for age and sex. Model 3: additionally adjusted for HbA_1c_. Model 4: additionally adjusted for mean arterial blood pressure and heart rate in case of cf-PWV. Model 5: additionally adjusted for body mass index, smoking status, physical activity, Mediterranean diet score, use of antihypertensive and lipid-modifying drugs, fasting triglycerides, and total-to-HDL cholesterol levels.

| **Model** | **B (95%CI)** | ***P* value** |
| --- | --- | --- |
| *Carotid femoral pulse wave velocity (cf-PWV), m/s (n=1,954)* | | |
| Crude | 0.011 (0.009; 0.013) | < 0.001 |
| Model 2 | 0.007 (0.005; 0.009) | < 0.001 |
| Model 3 | 0.005 (0.003; 0.007) | < 0.001 |
| Model 4* | 0.004 (0.001; 0.006) | 0.001 |
| Model 5 | 0.003 (0.001; 0.005) | 0.010 |
| *Carotid distensibility coefficient (carDC), 10^-3^/kPa (n=1,978)* | | |
| Crude | -0.020 (-0.025; -0.014) | < 0.001 |
| Model 2 | -0.011 (-0.016; -0.006) | < 0.001 |
| Model 3 | -0.005 (-0.011; 0.000) | 0.071 |
| Model 4* | -0.003 (-0.008; 0.002) | 0.274 |
| Model 5 | -0.002 (-0.007; 0.003) | 0.498 |
| *Carotid intima-media thickness (cIMT),* *µm (n=1,973)* | | |
| Crude | 0.204 (0.040; 0.368) | 0.015 |
| Model 2 | -0.062 (-0.221; 0.097) | 0.445 |
| Model 3 | -0.154 (-0.335; 0.026) | 0.094 |
| Model 4† | -0.203 (-0.383; -0.023) | 0.027 |
| Model 5 | -0.193 (-0.378; -0.008) | 0.041 |
| *Mean circumferential wall stress (CWS_mean_), kPa (n=1,870)* | | |
| Crude | 0.029 (0.017; 0.041) | < 0.001 |
| Model 2 | 0.022 (0.010; 0.034) | < 0.001 |
| Model 3 | 0.022 (0.008; 0.036) | 0.002 |
| Model 4‡ | 0.019 (0.005; 0.032) | 0.006 |
| Model 5 | 0.013 (-0.001; 0.026) | 0.069 |
| *Pulsatile circumferential wall stress (CWS_puls_), kPa (n=1,849)* | | |
| Crude | 0.027 (0.017; 0.036) | < 0.001 |
| Model 2 | 0.017 (0.008; 0.027) | < 0.001 |
| Model 3 | 0.018 (0.007; 0.029) | 0.001 |
| Model 4* | 0.012 (0.002; 0.021) | 0.019 |
| Model 5 | 0.007 (-0.003; 0.017) | 0.173 |
| *Retinal arteriolar average dilatation (n=1,591)* | | |
| Crude | -0.005 (-0.008; -0.001) | 0.005 |
| Model 2 | -0.004 (-0.007; 0.000) | 0.040 |
| Model 3 | -0.001 (-0.005; 0.003) | 0.536 |
| Model 4† | -0.001 (-0.005; 0.003) | 0.476 |
| Model 5 | 0.000 (-0.004; 0.004) | 0.910 |
| *Heat-induced skin hyperemia (n=1,134)* | | |
| Crude | -1.852 (-2.951; -0.754) | 0.001 |
| Model 2 | -0.896 (-2.002; 0.209) | 0.112 |
| Model 3 | -0.440 (-1.686; 0.805) | 0.488 |
| Model 4† | -0.544 (-1.797; 0.709) | 0.395 |
| Model 5 | -0.289 (-1.577; 1.000) | 0.661 |

**Additional file 1: Table S10. Multivariable-adjusted associations of percentage increase from baseline (IGP_percentage_) and arterial stiffness, arterial remodeling and microvascular function**

Regression coefficients (B) indicate the mean difference (95% confidence interval) associated with 1 unit (%) increase of percentage increase from baseline (IGPpercentage). Model 1: crude. Model 2: additionally adjusted for age and sex. Model 3: additionally adjusted for HbA_1c_. Model 4: additionally adjusted for mean arterial pressure (*), office systolic blood pressure (†) or carotid pulse pressure (‡) and heart rate in case of cf-PWV. Model 5: additionally adjusted for body mass index, smoking status, physical activity, Mediterranean diet score, use of antihypertensive and lipid-modifying drugs, fasting triglycerides, and total-to-HDL cholesterol levels.

**Additional file 1: Table S11. Multivariable-adjusted associations of incremental glucose peak (IGP) and arterial stiffness after adjustment for alternative blood pressure measurements**

| **Model** | **B (95%CI)** | ***P* value** |
| --- | --- | --- |
| *Carotid-femoral pulse wave velocity (cf-PWV), m/s (n=1,950)* | | |
| Crude | 0.171 (0.142; 0.199) | < 0.001 |
| Model 2 | 0.109 (0.082; 0.136) | < 0.001 |
| Model 3 | 0.097 (0.062; 0.133) | < 0.001 |
| Model 4* | 0.048 (0.014; 0.082) | 0.005 |
| Model 5 | 0.041 (0.006; 0.076) | 0.021 |
| *Carotid-femoral pulse wave velocity (cf-PWV), m/s (n=1,751)* | | |
| Crude | 0.170 (0.140; 0.200) | < 0.001 |
| Model 2 | 0.110 (0.081; 0.138) | < 0.001 |
| Model 3 | 0.100 (0.062; 0.138) | < 0.001 |
| Model 4† | 0.074 (0.039; 0.110) | < 0.001 |
| Model 5 | 0.061 (0.024; 0.098) | 0.001 |
| *Carotid distensibility coefficient (carDC), 10^-3^/kPa (n=1,974)* | | |
| Crude | -0.313 (-0.388; -0.239) | < 0.001 |
| Model 2 | -0.186 (-0.255; -0.117) | < 0.001 |
| Model 3 | -0.092 (-0.184; -0.001) | 0.048 |
| Model 4* | -0.029 (-0.119; 0.060) | 0.521 |
| Model 5 | -0.021 (-0.113; 0.070) | 0.648 |
| *Carotid distensibility coefficient (carDC), 10^-3^/kPa (n=1,769)* | | |
| Crude | -0.274 (-0.353; -0.195) | < 0.001 |
| Model 2 | -0.153 (-0.227; -0.079) | < 0.001 |
| Model 3 | -0.061 (-0.158; 0.035) | 0.214 |
| Model 4† | -0.054 (-0.148; 0.041) | 0.266 |
| Model 5 | -0.030 (-0.128; 0.067) | 0.539 |

Regression coefficients (B) indicate the mean difference (95% confidence interval) associated with 1 unit (mmol/L) increase of IGP. Model 1: crude. Model 2: additionally adjusted for age and sex. Model 3: additionally adjusted for HbA_1c_. Model 4: additionally adjusted for office systolic blood pressure (*) or ambulatory 24-h systolic blood pressure (†) and heart rate in case of cf-PWV. Model 5: additionally adjusted for body mass index, smoking status, physical activity, Mediterranean diet score, use of antihypertensive and lipid-modifying drugs, fasting triglycerides, and total-to-HDL cholesterol levels.

**Additional file 1: Table S12. Multivariable-adjusted associations of incremental glucose peak (IGP) and arterial remodeling after adjustment for alternative blood pressure measurements**

| **Model** | **B (95%CI)** | ***P* value** |
| --- | --- | --- |
| *Carotid intima-media thickness (cIMT), µm (n=1,769)* | | |
| Crude | 3.315 (0.947; 5.683) | 0.006 |
| Model 2 | -1.085 (-3.409; 1.239) | 0.360 |
| Model 3 | -3.088 (-6.147; -0.029) | 0.048 |
| Model 4* | -3.287 (-6.310; -0.265) | 0.033 |
| Model 5 | -3.126 (-6.269; 0.017) | 0.051 |
| *Carotid intima-media thickness (cIMT), µm (n=1,974)* | | |
| Crude | 4.137 (1.919; 6.355) | < 0.001 |
| Model 2 | -0.124 (-2.309; 2.061) | 0.912 |
| Model 3 | -2.136 (-5.029; 0.758) | 0.148 |
| Model 4† | -2.455 (-5.345; 0.434) | 0.096 |
| Model 5 | -2.193 (-5.198; 0.813) | 0.153 |
| *Mean circumferential wall stress (CWS_mean_), kPa (n=1,765)* | | |
| Crude | 0.443 (0.277; 0.609) | < 0.001 |
| Model 2 | 0.33 (0.16; 0.501) | < 0.001 |
| Model 3 | 0.404 (0.180; 0.628) | < 0.001 |
| Model 4* | 0.383 (0.165; 0.602) | 0.001 |
| Model 5 | 0.268 (0.043; 0.492) | 0.019 |
| *Mean circumferential wall stress (CWS_mean_), kPa (n=1,969)* | | |
| Crude | 0.408 (0.251; 0.564) | < 0.001 |
| Model 2 | 0.288 (0.128; 0.449) | < 0.001 |
| Model 3 | 0.385 (0.172; 0.598) | < 0.001 |
| Model 4‡ | 0.248 (0.040; 0.457) | 0.020 |
| Model 5 | 0.162 (-0.053; 0.377) | 0.139 |
| *Pulsatile circumferential wall stress (CWS_puls_), kPa (n=1,767)* | | |
| Crude | 0.372 (0.235; 0.509) | < 0.001 |
| Model 2 | 0.227 (0.088; 0.366) | 0.001 |
| Model 3 | 0.283 (0.101; 0.464) | 0.002 |
| Model 4* | 0.250 (0.077; 0.423) | 0.005 |
| Model 5 | 0.163 (-0.016; 0.342) | 0.074 |
| *Pulsatile circumferential wall stress (CWS_puls_), kPa (n=1,848)* | | |
| Crude | 0.398 (0.268; 0.529) | < 0.001 |
| Model 2 | 0.256 (0.123; 0.389) | < 0.001 |
| Model 3 | 0.333 (0.158; 0.509) | < 0.001 |
| Model 4‡ | 0.155 (-0.011; 0.320) | 0.067 |
| Model 5 | 0.099 (-0.073; 0.270) | 0.259 |

Regression coefficients (B) indicate the mean difference (95% confidence interval) associated with 1 unit (mmol/L) increase of IGP. Model 1: crude. Model 2: additionally adjusted for age and sex. Model 3: additionally adjusted for HbA_1c_. Model 4: additionally adjusted for ambulatory 24-hour systolic blood pressure (*), mean arterial pressure (†), or office systolic blood pressure (‡). Model 5: additionally adjusted for body mass index, smoking status, physical activity, Mediterranean diet score, use of antihypertensive and lipid-modifying drugs, fasting triglycerides, and total-to-HDL cholesterol levels.

**Additional file 1: Table S13. Multivariable-adjusted associations of incremental glucose peak (IGP) and microvascular function after adjustment for alternative blood pressure measurements**

| **Model** | **B (95%CI)** | ***P* value** |
| --- | --- | --- |
| *Retinal arteriolar average dilatation (n=1,422)* | | |
| Crude | -0.091 (-0.14; -0.043) | < 0.001 |
| Model 2 | -0.075 (-0.125; -0.025) | 0.003 |
| Model 3 | -0.040 (-0.106; 0.026) | 0.238 |
| Model 4 | -0.040 (-0.106; 0.026) | 0.231 |
| Model 5 | -0.023 (-0.092; 0.046) | 0.512 |
| *Heat-induced skin hyperemia (n=1,000)* | | |
| Crude | -27.164 (-42.764; -11.563) | 0.001 |
| Model 2 | -13.087 (-28.994; 2.819) | 0.107 |
| Model 3 | -5.924 (-27.064; 15.216) | 0.583 |
| Model 4 | -5.654 (-26.806; 15.499) | 0.600 |
| Model 5 | -0.237 (-22.360; 21.886) | 0.983 |

Regression coefficients (B) indicate the mean difference (95% confidence interval) associated with 1 unit (mmol/L) increase of IGP. Model 1: crude. Model 2: additionally adjusted for age and sex. Model 3: additionally adjusted for HbA_1c_. Model 4: additionally adjusted for ambulatory 24-hour systolic blood pressure. Model 5: additionally adjusted for body mass index, smoking status, physical activity, Mediterranean diet score, use of antihypertensive and lipid-modifying drugs, fasting triglycerides, and total-to-HDL cholesterol levels.

**Additional file 1: Table S14. Multivariable-adjusted associations of time to glucose peak and arterial stiffness, arterial remodeling and microvascular function**

| **Model** | **B (95%CI)** | ***P* value** |
| --- | --- | --- |
| *Carotid femoral pulse wave velocity (cf-PWV), m/s (n=1,954)* | | |
| Crude | 0.018 (0.015; 0.022) | < 0.001 |
| Model 2 | 0.011 (0.008; 0.008) | < 0.001 |
| Model 3 | 0.009 (0.005; 0.005) | < 0.001 |
| Model 4^*^ | 0.006 (0.002; 0.009) | 0.001 |
| Model 5 | 0.005 (0.001; 0.008) | 0.007 |
| *Carotid distensibility coefficient (carDC), 10^-3^/kPa (n=1,978)* | | |
| Crude | -0.030 (-0.039; -0.022) | < 0.001 |
| Model 2 | -0.014 (-0.022; -0.006) | < 0.001 |
| Model 3 | -0.004 (-0.013; 0.005) | 0.425 |
| Model 4^*^ | 0.004 (-0.005; 0.012) | 0.402 |
| Model 5 | 0.006 (-0.002; 0.015) | 0.131 |
| *Carotid intima-media thickness (cIMT),* *µm (n=1,973)* | | |
| Crude | 0.501 (0.246; 0.736) | < 0.001 |
| Model 2 | 0.031 (-0.219; 0.282) | 0.807 |
| Model 3 | -0.081 (-0.368; 0.206) | 0.579 |
| Model 4^†^ | -0.189 (-0.475; 0.098) | 0.196 |
| Model 5 | -0.140 (-0.433; 0.152) | 0.348 |
| *Mean circumferential wall stress (CWS_mean_), kPa (n=1,869)* | | |
| Crude | 0.048 (0.029; 0.067) | < 0.001 |
| Model 2 | 0.036 (0.017; 0.055) | < 0.001 |
| Model 3 | 0.037 (0.015; 0.059) | 0.001 |
| Model 4^‡^ | 0.029 (0.008; 0.051) | 0.007 |
| Model 5 | 0.015 (-0.006; 0.037) | 0.162 |
| *Pulsatile circumferential wall stress (CWS_puls_), kPa (n=1,849)* | | |
| Crude | 0.043 (0.028; 0.058) | < 0.001 |
| Model 2 | 0.027 (0.012; 0.042) | 0.001 |
| Model 3 | 0.029 (0.011; 0.046) | 0.001 |
| Model 4^*^ | 0.011 (-0.004; 0.027) | 0.155 |
| Model 5 | 0.005 (-0.011; 0.021) | 0.546 |
| *Retinal arteriolar average dilatation (n=1,591)* | | |
| Crude | -0.011 (-0.016; -0.005) | < 0.001 |
| Model 2 | -0.009 (-0.014; -0.028) | 0.001 |
| Model 3 | -0.007 (-0.012; -0.001) | 0.029 |
| Model 4^†^ | -0.007 (-0.013; -0.001) | 0.022 |
| Model 5 | -0.006 (-0.012; 0.001) | 0.074 |
| *Heat-induced skin hyperemia (n=1,134)* | | |
| Crude | -3.181 (-4.867; -1.495) | < 0.001 |
| Model 2 | -1.521 (-3.236; 0.194) | 0.082 |
| Model 3 | -0.814 (-2.777; 1.149) | 0.416 |
| Model 4^†^ | -1.046 (-3.032; 0.940) | 0.302 |
| Model 5 | -1.044 (-3.080; 0.993) | 0.315 |

Regression coefficients (B) indicate the mean difference (95% confidence interval) associated with 1 unit (minute) increase of time to glucose peak. Model 1: crude. Model 2: additionally adjusted for age and sex. Model 3: additionally adjusted for HbA_1c_. Model 4: additionally adjusted for mean arterial blood pressure and heart rate in case of cf-PWV. Model 5: additionally adjusted for body mass index, smoking status, physical activity, Mediterranean diet score, use of antihypertensive and lipid-modifying drugs, fasting triglycerides, and total-to-HDL cholesterol levels.

**Additional file 1: Table S15. Fully adjusted associations of incremental glucose peak (IGP) and carotid-femoral pulse wave velocity (cf-PWV) stratified according to age tertiles**

| **Age tertiles** | **Model 5: B (95%CI)** | ***P* value** |
| --- | --- | --- |
| *Carotid-femoral pulse wave velocity (cf-PWV), m/s* | | |
| 1^st^ (n=627) | 0.032 (-0.021; 0.085) | 0.234 |
| 2^nd^ (n=707) | 0.034 (-0.020; 0.088) | 0.219 |
| 3^rd^ (n=620) | 0.101 (0.032; 0.169) | 0.004 |

Regression coefficients (B) indicate the mean difference (95% confidence interval) associated with 1 unit (mmol/L) increase of IGP. Model 5: fully adjusted model.

**Additional file 1: Table S16. Fully adjusted associations of incremental glucose peak (IGP) and carotid distensibility coefficient (carDC) stratified according to age tertiles**

| **Age tertiles** | **Model 5: B (95%CI)** | ***P* value** |
| --- | --- | --- |
| *Carotid destensibility coefficient (carDC), 10^-3^/kPa* | | |
| 1^st^ (n=634) | -0.120 (-0.306; 0.066) | 0.207 |
| 2^nd^ (n=712) | -0.070 (-0.205; 0.066) | 0.313 |
| 3^rd^ (n=632) | 0.071 (-0.067; 0.208) | 0.313 |

Regression coefficients (B) indicate the mean difference (95% confidence interval) associated with 1 unit (mmol/L) increase of IGP. Model 5: fully adjusted model.

**Additional file 1: Table S17. Fully adjusted associations of incremental glucose peak (IGP) and pulsatile circumferential wall stress (CWS_puls_) stratified according to age tertiles**

| **Age tertiles** | **Model 5: B (95%CI)** | ***P* value** |
| --- | --- | --- |
| *Pulsatile circumferential wall stress (CWS_puls_), kPa* | | |
| 1^st^ (n=525) | -0.135 (-0.405; 0.135) | 0.326 |
| 2^nd^ (n=594) | 0.170 (-0.112; 0.451) | 0.237 |
| 3^rd^ (n=514) | 0.270 (-0.027; 0.566) | 0.075 |

Regression coefficients (B) indicate the mean difference (95% confidence interval) associated with 1 unit (mmol/L) increase of IGP. Model 5: fully adjusted model.
